# Region-specific variation in the properties of skeletal adipocytes reveals regulated and constitutive marrow adipose tissues

**DOI:** 10.1038/ncomms8808

**Published:** 2015-08-06

**Authors:** Erica L. Scheller, Casey R. Doucette, Brian S. Learman, William P. Cawthorn, Shaima Khandaker, Benjamin Schell, Brent Wu, Shi-Ying Ding, Miriam A. Bredella, Pouneh K. Fazeli, Basma Khoury, Karl J. Jepsen, Paul F. Pilch, Anne Klibanski, Clifford J. Rosen, Ormond A. MacDougald

**Affiliations:** 1 Departments of Molecular and Integrative Physiology and Internal Medicine, University of Michigan, Ann Arbor, Michigan 48105, USA; 2 Center for Clinical and Translational Research, Maine Medical Center Research Institute, Scarborough, Maine 04074, USA; 3 Department of Biochemistry, Boston University School of Medicine, Boston, Massachusetts 02118, USA; 4 Department of Radiology, Massachusetts General Hospital and Harvard Medical School, Boston, Massachusetts 02114, USA; 5 Neuroendocrine Unit, Massachusetts General Hospital and Harvard Medical School, Boston, Massachusetts 02114, USA; 6 Department of Orthopaedic Surgery, University of Michigan, Ann Arbor, Michigan 48109, USA

## Abstract

Marrow adipose tissue (MAT) accumulates in diverse clinical conditions but remains poorly understood. Here we show region-specific variation in MAT adipocyte development, regulation, size, lipid composition, gene expression and genetic determinants. Early MAT formation in mice is conserved, whereas later development is strain dependent. Proximal, but not distal tibial, MAT is lost with 21-day cold exposure. Rat MAT adipocytes from distal sites have an increased proportion of monounsaturated fatty acids and expression of *Scd1/Scd2*, *Cebpa* and *Cebpb*. Humans also have increased distal marrow fat unsaturation. We define proximal ‘regulated’ MAT (rMAT) as single adipocytes interspersed with active haematopoiesis, whereas distal ‘constitutive’ MAT (cMAT) has low haematopoiesis, contains larger adipocytes, develops earlier and remains preserved upon systemic challenges. Loss of rMAT occurs in mice with congenital generalized lipodystrophy type 4, whereas both rMAT and cMAT are preserved in mice with congenital generalized lipodystrophy type 3. Consideration of these MAT subpopulations may be important for future studies linking MAT to bone biology, haematopoiesis and whole-body metabolism.

## Abstract (web summary)

Bone marrow contains adipocytes, which have been thought to form one type of marrow adipose tissue (MAT). Here, the authors identify two MAT subpopulations in mice and humans—‘regulated’ and ‘constitute’ MAT—which show distinct phenotypic and cellular traits, and respond differently to cold exposure.

## Graphical abstract

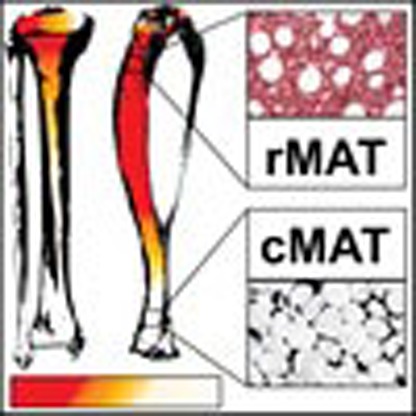

Marrow adipose tissue (MAT) is a functionally distinct adipose depot, located within the skeleton, with the potential to contribute to both local and systemic metabolism^1^,^2^. Further accumulation of MAT occurs in a diverse range of clinical conditions including osteoporosis, ageing, gonadal dysfunction, type 1 diabetes and anorexia^2^,^3^. MAT formation is also induced with therapeutic interventions including radiation, chemotherapy, glucocorticoids and thiazolidinediones^1^,^3^. Despite these clinical findings, the regulation and function of MAT remains largely unclear.

In many cases, MAT accumulation has been correlated with low bone mineral density, decreased bone formation and bone loss (reviewed in ref. 2). However, the presence of a direct relationship between MAT and bone remains controversial. For example, despite a clear correlation, increased MAT is not necessary for bone loss at the proximal tibia in rodent models of type 1 diabetes or ovariectomy-induced osteopenia^4^,^5^,^6^. In addition, histomorphometric studies in rats demonstrate that sites of high MAT have decreased ovariectomy-induced trabecular bone loss, with trabecular width in rat tibial metaphyses being greater at sites of high MAT (distal tibia) than at sites of low MAT (proximal tibia)^7^,^8^,^9^. The hypothesis that MAT is necessary for skeletal equilibrium is also supported by phenotypes of patients with congenital generalized lipodystrophy (CGL). A high proportion of patients with CGL1 or CGL2 (who lack MAT) develop pathological osteosclerosis and skeletal cysts between ages 10 and 20 years—the time in humans when MAT generally undergoes robust formation in a developmentally defined pattern in the affected skeletal regions^2^. In contrast, those with CGL3 or CGL4 (who retain MAT) fail to develop this pathology. These apparent contradictions emphasize the complex, context-specific relationship between MAT and bone, and likely the relationship between MAT and peripheral metabolism^1^,^10^.

Although it is generally assumed that all marrow adipocytes are equivalent, a study by Tavassoli^11^ in 1976 suggested that characteristics of red marrow adipocytes may differ to those of adipocytes within yellow marrow. In humans, formation of adipocytes within the yellow marrow occurs at or slightly before birth, regardless of prematurity, and accelerates between 4 and 8 weeks of age^2^,^12^. Early MAT formation occurs in distal skeletal regions including the hands, feet, distal tibia and tail (in rodents). Histologically, once this early MAT matures, the densely packed adipocytes resemble peripheral white adipose tissue (WAT) and are relatively devoid of active haematopoiesis. For the purposes of discussion in this paper, we define these areas as constitutive MAT (cMAT). After the initial peak, MAT accumulation continues in areas of red, haematopoietic marrow throughout life^13^. We refer to this population as regulated MAT (rMAT) and define it histologically as single adipocytes interspersed with sites of active haematopoiesis. It is important to note that, especially in larger species, both histological patterns may exist side by side. In rats and mice, however, these regions appear to be more spatially distinct.

We hypothesized that the later-forming rMAT adipocytes would have characteristics distinct from the cMAT adipocytes that arise early in development. Herein, we address this hypothesis using mouse models to examine MAT formation and regulation during development and with cold exposure; lipidomics and proton magnetic resonance (MR) spectroscopy (^1^H-MRS) to measure MAT lipid composition in rats and humans; MAT isolated from rats to quantify molecular differences in gene expression; and CGL3 and CGL4 mouse models that reveal a genetic basis for development of distinct rMAT and cMAT subpopulations. In sum, this evidence distinguishes rMAT from cMAT—a fundamental finding that may help to explain previous inconsistencies in the literature and inform future research on the relationship between MAT, bone, haematopoiesis and whole-body metabolism.

## Results

### Strain-specific MAT development in mice

**Figure 1:**
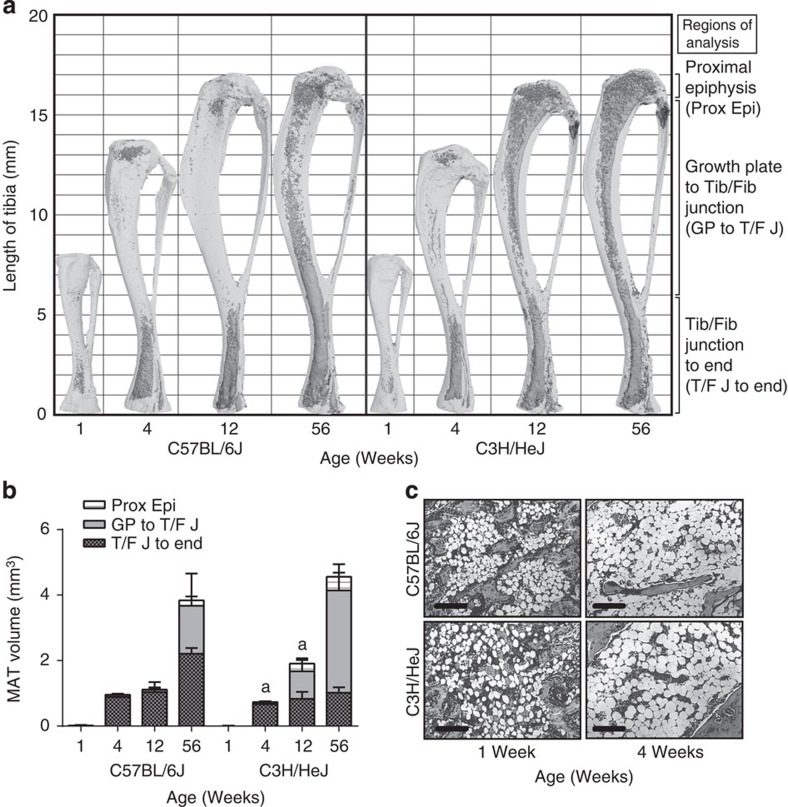
Quantification of MAT development in C57BL/6J and C3H/HeJ mice from 1 to 56 weeks of age. (**a**) Osmium-stained tibiae were scanned by μCT and were reconstructed with the decalcified bone overlaid. Representative images of the data are presented in (**b**). Marrow fat is dark grey and bone is light grey. (**b**) Region-specific quantification of MAT volume (biological replicate *N*=5 (1 week old), 7 (4 weeks old), 9 (C3H 12 weeks old) and 11 (B6 12- and 56 weeks old)). Regions as defined in **a** include the proximal epiphysis (Prox Epi), the growth plate to the tibia/fibula (Tib/Fib) junction (GP to T/F J) and the tibia/fibula junction to the end of the bone (T/F J to end). ^a^Two-tailed *t*-test, *P*<0.05 for total tibial MAT volume compared between strains at a given age. (**c**) Representative histology of caudal vertebrae (biological replicate *N* = 5), × 10 objective (scale bars, 200 μm). All graphs represent mean ± s.d.

The postnatal development of MAT remains poorly characterized on a spatiotemporal level. We used osmium tetroxide staining to visualize and quantify MAT in the whole tibia of male C57BL/6J (B6) and C3H/HeJ (C3H) mice at 1, 4, 12 and 56 weeks of age (Fig. 1). At 1 and 4 weeks, the initial phase of MAT development was similar in both strains. In the distal tibia, MAT formation and maturation accelerated rapidly after birth until the marrow space filled with adipocytes at 4 weeks of age. The amount of MAT distal to the junction of the tibia and fibula was similar between B6 and C3H strains through 12 weeks and remained relatively stable until 56 weeks in C3H animals (Fig. 1a,b). A parallel pattern of development occurred in the caudal vertebrae of the tail, with mature MAT filling the marrow space by 4 weeks of age (Fig. 1c). At this time, MAT in the tail vertebrae matched the histological appearance of cMAT as defined above.

In contrast to the distal tibia and tail, rMAT within the middle and proximal tibia was highly variable in both volume and rate of development (Fig. 1a,b). By 12 weeks, MAT development diverged, with robust expansion in the proximal tibial marrow of C3H, but not B6, mice. Thus, C3H mice had nearly twice as much total MAT than B6 at this age. Surprisingly, by 56 weeks these differences in total MAT disappeared (Fig. 1b); however, the distribution of the cells within the tibia remained divergent, with C3H mice having increased MAT volume in the proximal regions of the tibia (Fig. 1b). These distinct developmental characteristics suggest discrete MAT populations, designated cMAT (distal tibia and tail vertebrae) and rMAT (mid- to proximal tibia).

**Figure 2:**
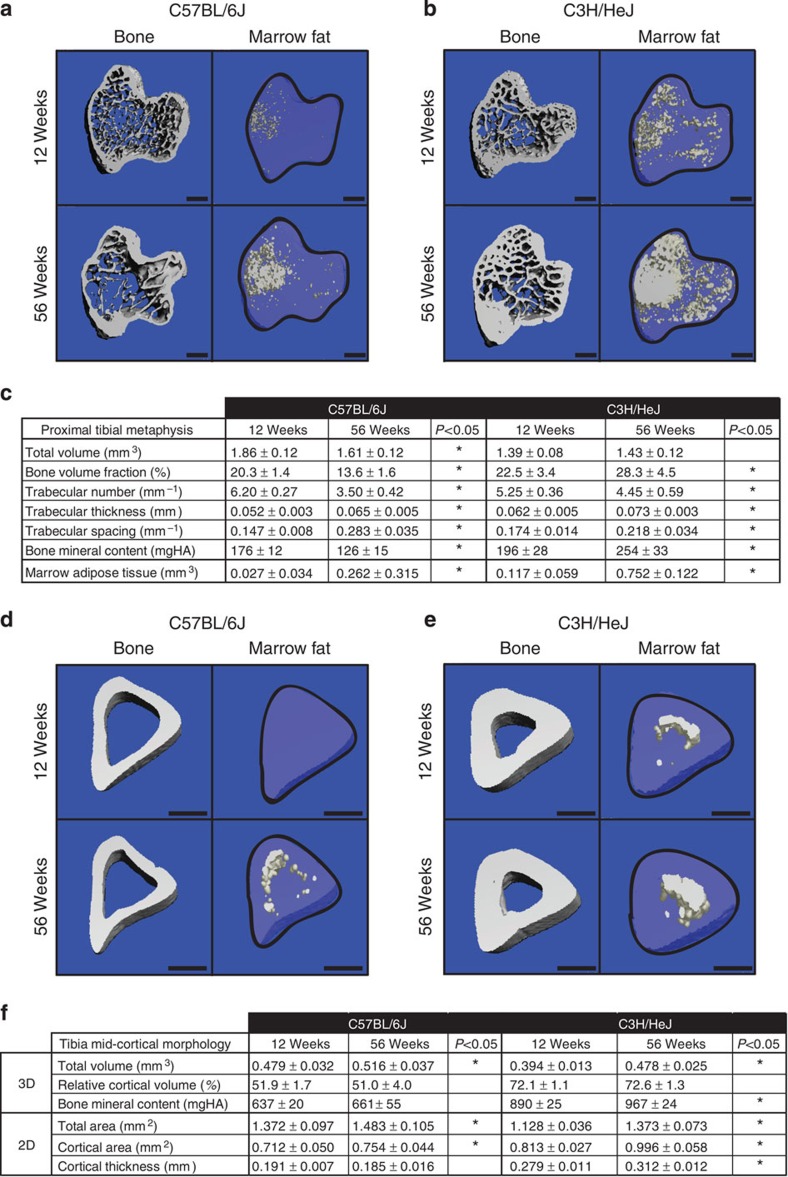
Trabecular and cortical development in C57BL/6J and C3H/HeJ mice at 12 and 56 weeks of age. (**a**,**b**) Representative images of the proximal tibial metaphysis both before decalcification and after osmium staining of the data presented in **c**. Marrow fat is in white. (**c**) Quantification of trabecular parameters and MAT volume in the proximal tibial metaphysis (biological replicate *N*=9 (C3H 12 weeks old) and 11 (B6 12- and 56 weeks old)). (**d**,**e**) Representative images of the mid-tibial diaphysis both before decalcification and after osmium staining of the data presented in panel **f**. Marrow fat is in white. (**f**) Quantification of cortical parameters (biological replicate *N*=9 (C3H 12 weeks old) and 11 (B6 12- and 56 weeks old)). Scale bars=500 μm. *Two-tailed *t*-test, *P*<0.05. All values represent mean±s.d.

To examine the developmental relationship between MAT and bone, we also analysed the tibiae of 12- and 56-week-old animals both before decalcification and again after osmium staining. Osmium-based localization of MAT in three dimensions demonstrated its asymmetric distribution within the tibial marrow cavity (Fig. 2a,b). In both B6 and C3H mouse strains, MAT accumulation with age in the proximal metaphysis occurred most robustly in the medial marrow space. In the mid-diaphysis, B6 MAT continued to approximate the medial endocortical surface, whereas C3H MAT closely followed the posterior cortex (Fig. 2d,e). Development of trabecular and cortical bone was similar to what has been reported previously (Fig. 2c,f; ref. 14). In addition to increases in MAT with age in both strains, trabecular number decreased and thickness increased. Thus, in the proximal metaphysis across the 12- and 56-week-old groups, MAT volume correlated negatively with trabecular number (linear regression B6, *P*=0.007; C3H, *P*=0.005) but positively with trabecular thickness (linear regression B6, *P*=0.010; C3H, *P*<0.001).

### Differential loss of MAT with cold exposure

Cold exposure in rodents elevates sympathetic tone and results in extensive remodelling of WAT, which enhances thermogenesis and allows maintenance of body temperature and survival at 4 °C (ref. 15). However, the response of MAT to cold temperatures is unknown. To quantify changes in MAT after 21-day cold exposure (4 °C), we analysed MAT in the whole tibia of male C3H mice at 12 and 56 weeks of age. The C3H strain was used based on the robust proportion of MAT in both proximal and distal regions of the tibia (Fig. 1), allowing for simultaneous analysis of rMAT and cMAT populations within the same bone.

**Figure 3:**
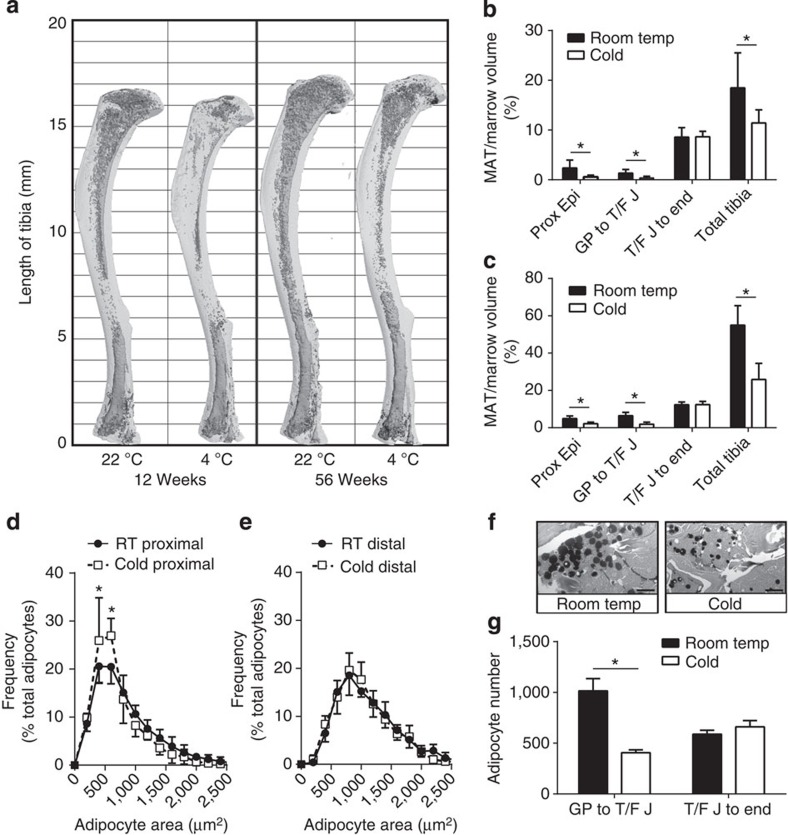
Differential loss of MAT with cold exposure. (**a**) Representative osmium-stained tibiae scanned with μCT of the data presented in **b** and **c**. Marrow fat in dark grey, decalcified bone overlaid in light grey. (**b**) Region-specific quantification of tibial MAT (as defined in Fig. 1a) from 12-week-old mice normalized to marrow volume (biological replicate *N*=11 (4 °C) and 11 (22 °C)). (**c**) Region-specific quantification of tibial MAT from 56-week-old mice normalized to marrow volume (biological replicate *N*=9 (4 °C) and 11 (22 °C)). (**d**) Adipocyte size distribution from the proximal tibial metaphysis (proximal) or (**e**) distal diaphysis below the tibia/fibula junction (distal) as measured by nanoCT in the 12-week-old mice (biological replicate *N* = 5). Histogram bin size 250. (**f**) Representative histology, based on quantification in **d**, of osmium-stained samples at the proximal tibia shows a decrease in adipocyte size. Scale bars, 50 μm. (**g**) Estimation of region-specific adipocyte number was performed by dividing the total adipocyte volume (from μCT) by the average adipocyte volume (nanoCT) in the proximal tibia (growth plate to tibia/fibula junction) and the distal tibia (tibia/fibula junction to the distal end). *(**b**,**c**,**g**) Two-tailed *t*-test, (**d**,**e**) two-way analysis of variance with Sidak’s multiple comparisons test, *P*<0.05. RT, room temperature. All graphs represent mean±s.d.

After cold exposure in 12-week-old mice, the amount of rMAT decreased by 76% in the tibial epiphysis and 71% in the proximal tibia, between the growth plate and the tibia/fibula junction (Fig. 3a,b). In 56-week-old mice, rMAT decreased by 56 and 71%, respectively (Fig. 3c). In contrast, cMAT in the distal tibia, below the fibular attachment, remained unchanged (Fig. 3a–c). MAT loss at the proximal tibial metaphysis was most prominent in the centre of the marrow space with a relative preservation of the adipocytes that were directly adjacent to the endocortical surface (Supplementary Fig. 1A,B). This is the reverse of the developmental pattern of proximal MAT accumulation (Fig. 2a,b). Despite the robust loss of rMAT, trabecular and cortical parameters in the tibia remained largely unchanged (Supplementary Fig. 1C,D); indeed, the only significant finding was a slight decrease in the relative cortical bone volume in the 12-week-old C3H mice.

For osmium-based MAT analysis, we use a micro-computed tomography (μCT) voxel size of 12 μm, which allows rough outlines of the marrow adipocytes to be observed (the average MAT cell diameter is 30–40 μm). However, at this resolution, μCT might be unable to detect more subtle changes in regions of densely packed adipocytes, such as those in the distal tibia. To test this, we re-scanned the bones from the 12-week-old mice at a voxel size of 2 μm using nano-computed tomography (nanoCT). The resolution of these scans was sufficient to clearly identify individual adipocytes (Supplementary Fig. 2). Using a digital histology approach, we quantified adipocytes sizes in two-dimensional nanoCT DICOM slices (Supplementary Fig. 2; ref. 16). To determine the adipocyte size distribution, we measured the two-dimensional area of 300–400 individual adipocytes in the proximal tibial metaphysis and at the midpoint of the distal tibia (Supplementary Fig. 2). Consistent with our μCT results for total MAT volume, adipocytes in the proximal tibia decreased in size, whereas those in the distal tibia remained unchanged (Fig. 3d–f). This confirmed our μCT interpretation and the validity of the osmium/μCT method for total MAT volume quantification, even in adipocyte-dense regions such as the distal tibia^17^. Together, the μCT and nanoCT data revealed that in response to cold exposure, proximal rMAT adipocytes decrease in both size and number, whereas the adipocytes in the distal tibia are unchanged (Fig. 2g).

### Average adipocyte size of rMAT and cMAT adipocytes

**Figure 4:**
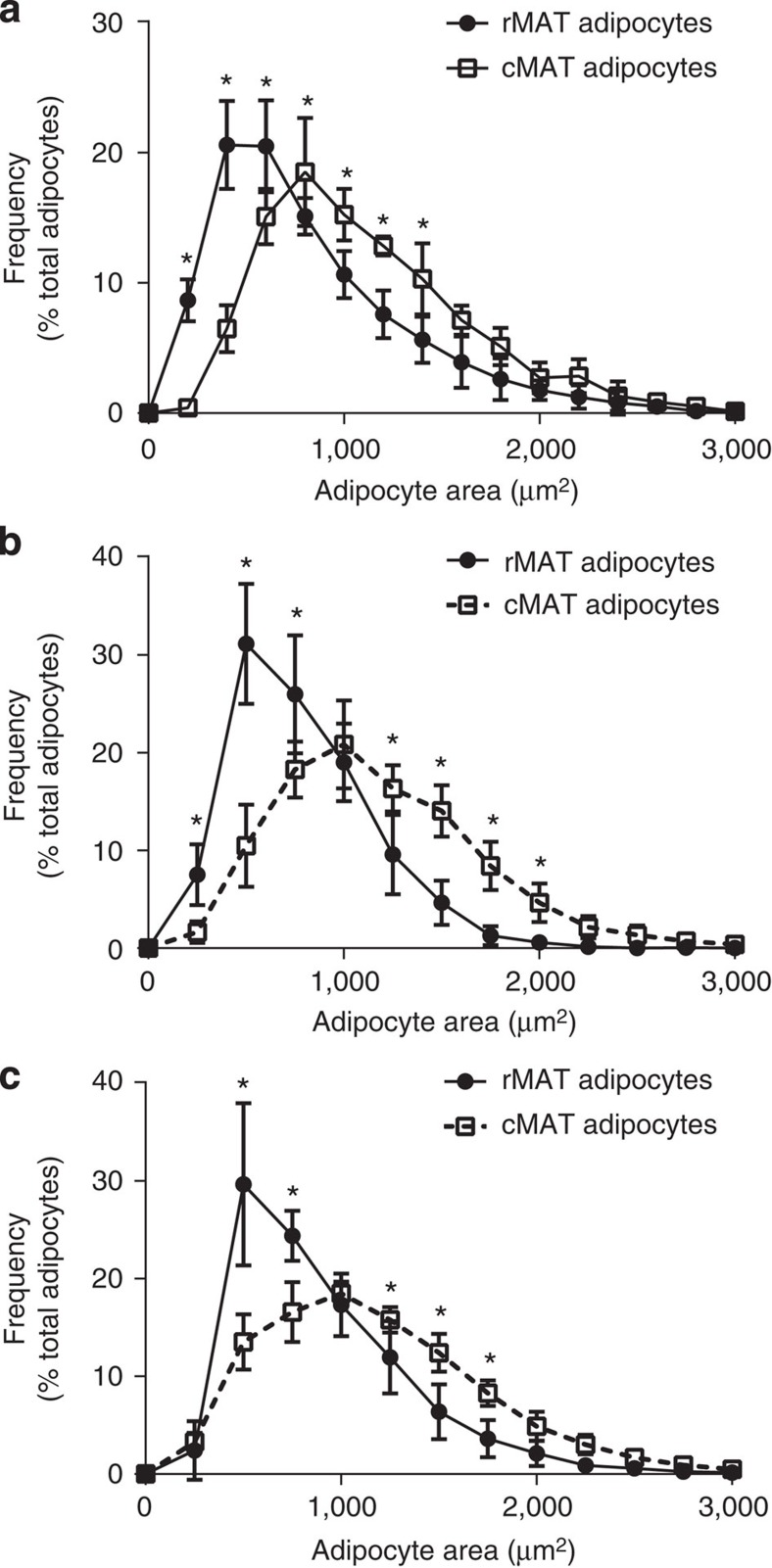
Quantification of rMAT and cMAT adipocyte size. Adipocyte size quantification from (**a**) rMAT in the proximal tibia and cMAT in the distal tibia of 12-week-old C3H mice (biological replicate *N*=5), (**b**) rMAT in the proximal tibia and cMAT in the caudal vertebrae of 16-week-old male Sprague–Dawley rats (biological replicate *N*=12), and (**c**) rMAT in the mid-tibia and cMAT in the caudal vertebrae of 19-week-old female Sprague–Dawley rats (biological replicate *N*=5 rMAT and 6 cMAT). *Two-way analysis of variance with Sidak’s multiple comparisons test, *P*<0.05. All graphs represent mean±s.d.

Adipocyte size is a parameter that has historically been used to track metabolic responsiveness of individual cells. Analysis of the 12-week-old C3H animals at room temperature revealed that cMAT adipocytes are significantly larger than rMAT adipocytes (Fig. 4a), with average diameters of 37.8±1.2 and 32.5±2.4 μm, respectively (two-tailed *t*-test, *P*=0.002). This 16% increase in cMAT adipocyte diameter extrapolates to an estimated 54.6% increase in cMAT adipocyte volume. cMAT adipocytes were also larger in rats (Fig. 4b,c), with cMAT adipocytes in tail vertebrae being 24 or 17% larger in diameter than tibial rMAT adipocytes in males or females, respectively (male cMAT versus rMAT, 38.9±1.9 versus 31.4±1.6 μm, two-tailed *t*-test, *P*<0.001; female cMAT versus rMAT, 38.9±1.6 versus 33.1±3.2 μm, two-tailed *t*-test, *P*=0.003). The cell size distributions for each group are presented as histograms in Fig. 4.

### Region-specific fatty-acid content of MAT

The mechanisms underlying site-specific regulation of marrow adipocytes with cold exposure could be related to differences in the local microenvironment and/or between adipocyte subpopulations. With the exception of Tavassoli^11^, previous work on marrow fat has assumed that all MAT adipocytes are equivalent. To begin testing the validity of this assumption and thus determine whether the microenvironment is the sole mediator, we characterized the lipidomic profile of the proximal rMAT and distal cMAT adipocytes.

**Figure 5:**
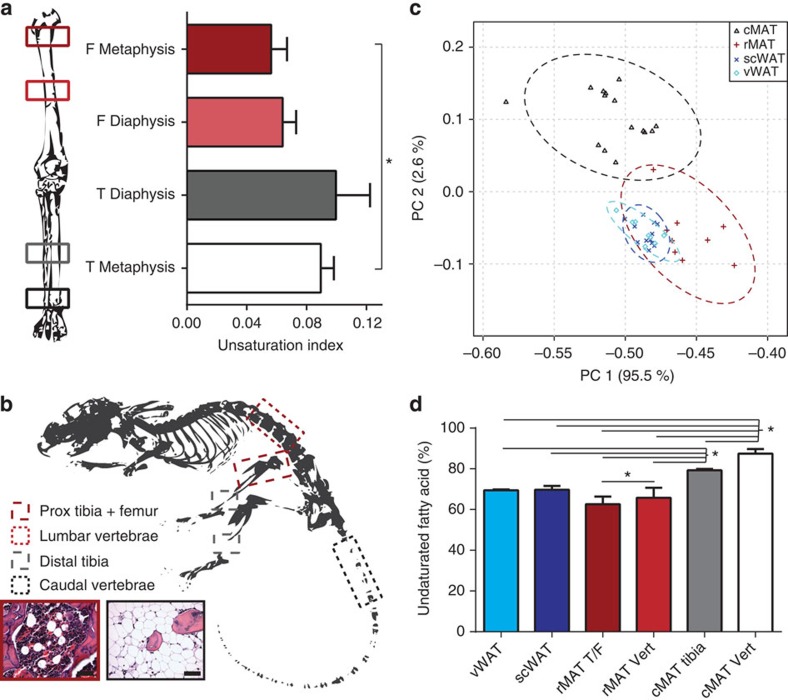
Region-specific lipid saturation of human and rat MAT. (**a**) Marrow unsaturation at four sites in the human leg was compared using ^1^H-MRS. Marrow at the metaphysis of the distal tibia (T) had a higher unsaturation index than marrow in the proximal femur (F) (biological replicate *N*=5). Mean±s.e. (**b**) Adipocytes were isolated from four regions of the rat skeleton. Red outlines indicate rMAT sites including femur/proximal (Prox) tibia and lumbar vertebrae. Grey and black outlines indicate cMAT sites including distal tibia and caudal vertebrae. Histology of intact rat rMAT and cMAT before adipocyte isolation representative of the animals from Fig. 4b (biological replicate *N*=12). Objective × 40, scale bars, 50 μm. (**c**) Principal components analysis of normalized fatty acids from three independent experiments (23 fatty acids and 44 unique biological samples). Raw data presented in Supplementary Data 1. Visceral WAT (vWAT) includes 5 gonadal and 3 perirenal; subcutaneous WAT (scWAT) includes 12 inguinal; rMAT includes 3 lumbar vertebrae and 6 femur/proximal tibia; cMAT includes 3 distal tibia and 12 caudal vertebrae (samples were derived from 13 unique animals). Dashed line = 95% confidence interval. (**d**) Proportion of fatty acids with one or more double bonds relative to total lipid in adipocytes from perirenal visceral WAT (vWAT), inguinal scWAT, rMAT from femur/proximal tibia (rMAT T/F), rMAT from lumbar vertebrae (rMAT Vert), cMAT from distal tibia (cMAT tibia) and cMAT from tail vertebrae (cMAT Vert). Representative data presented as mean±s.d. (as presented, biological replicate *N*=3). Experiment repeated with similar results in three animal cohorts with a total of 44 samples from 13 rats as outlined in Supplementary Data 1. *One-way analysis of variance with Tukey’s multiple comparisons test, *P*<0.05.

We started with lipidomics because the work of Tavassoli^11^ suggests that marrow adipocytes may have a region-specific lipid composition. In addition, our ‘marrow fat consortium’ group previously developed techniques to estimate the lipid unsaturation of MAT in the human skeleton using ^1^H-MRS^18^ (Supplementary Fig. 3). We applied this method to measure marrow lipid unsaturation in four regions of the human appendicular skeleton, including the femur (proximal metaphysis and mid-diaphysis) and the tibia (mid-diaphysis and distal metaphysis). We found that, in humans, the distal tibia had an increased unsaturation index relative to the proximal femur (Fig. 5a), implying that distal marrow adipocytes contain more unsaturated lipids than those in proximal/central skeletal regions.

Since the human model relies on indirect analysis of intact marrow, we developed a modified collagenase digestion protocol to purify adipocytes from the rat bone marrow for direct lipidomic analyses^19^. Adipocytes from WAT (perirenal, gonadal and inguinal) were used as a control. The rMAT regions included the femur/proximal tibia and lumbar vertebrae, whereas the cMAT regions included the distal tibia and caudal vertebrae (Fig. 5b). Adipocytes were isolated from a diverse population of rats including (experiment no. 1) 1-year-old female high-capacity runner rats^20^, (experiment no. 2) 16-week-old male Sprague–Dawley rats and (experiment no. 3) 8-month-old female Sprague–Dawley rats. After isolating adipocytes, we extracted total lipid with methanol–choloroform and then used gas chromatography (GC) for lipidomic analysis of esterified fatty acids.

In the adipocyte, the vast majority of fatty acids are derived from triacylglycerols with minor contributions from species such as phospholipids. Palmitate, stearate and their unsaturated derivatives were the most common—accounting for >90% of the total lipid. To standardize between experiments, we expressed each fatty-acid subtype as a per cent of the total lipid. The raw data for all experiments are presented in this format as Supplementary Data 1. This standardized data set was used to perform principal component analysis of the 23 fatty-acid subtypes across three independent experiments. In total, 44 unique lipidomic profiles of purified adipocytes from MAT (8 rMAT and 15 cMAT), visceral WAT (5 gonadal and 3 perirenal) and subcutaneous WAT (scWAT; 12 inguinal) were compared (Supplementary Data 1). Despite the diversity in the animal cohorts, all forms of WAT were tightly clustered while there was a clear separation of cMAT from rMAT and WAT (Fig. 5c). Consistent with the human data, the per cent of unsaturated fatty acids relative to total lipid was highest in the cMAT adipocytes purified from the distal tibia and the tail vertebrae (Fig. 5d).

**Figure 6:**
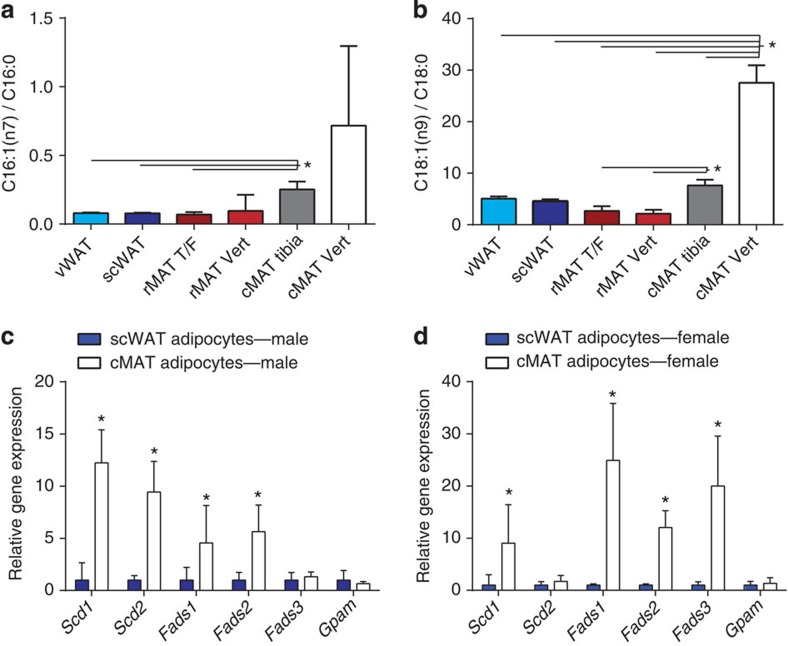
Gene expression of desaturases in isolated adipocytes. (**a**) Proportion of C16:1n7-palmitoleate relative to C16:0-palmitate. (**b**) The proportion of C18:1n9-oleate relative to C18:0-stearate. Representative data presented as mean±s.d. (as presented, biological replicate *N*=3). Repeated with similar results in three animal cohorts with samples from 13 total rats as detailed in Supplementary Data 1. Transcript expression in isolated constitutive MAT (cMAT) and subcutaneous WAT (scWAT) adipocytes normalized to scWAT from (**c**) 16-week-old male Sprague–Dawley rats (biological replicate *N*=6 cMAT (two animals pooled per sample) and *N*=12 scWAT) and (**d**) 8-month-old female Sprague–Dawley rats (biological replicate *N*=3 cMAT (two animals pooled per sample) and *N*=5 scWAT). Presented as mean±s.d. *(**a**,**b**) One-way analysis of variance with Tukey’s multiple comparisons test, (**c**,**d**) two-tailed *t*-test, *P*<0.05.

The increased proportion of unsaturated fatty acids in the rat cMAT adipocytes and separation from rMAT/WAT adipocytes on the principal component plot was primarily driven by decreases in palmitate and stearate, and corresponding increases in their monounsaturated derivatives palmitoleate and oleate (Supplementary Data 1). This resulted in a robust increase in the monounsaturated-to-saturated ratio for these fatty acids (Fig. 6a,b). This change was greater in cMAT adipocytes from the tail vertebrae when compared with the cMAT from the distal tibia, indicating that the distal tibia may be a region of mixed MAT. Consistent with the increased proportion of the unsaturated fatty acids palmitoleate and oleate, expression of stearoyl-CoA desaturase-1 (*Scd1*) was elevated in both male and female cMAT adipocytes relative to adipocytes isolated from scWAT (Fig. 6c,d). Elevated expression of desaturases including *Fads1* and *Fads2* was also noted in both males and females, with inconsistent elevations in *Scd2* (males only) and *Fads3* (females only; Fig. 6c,d). Expression of mitochondrial glycerol-3-phosphate acyltransferase (*Gpam*), an enzyme that preferentially incorporates saturated fatty acids during synthesis of glycerolipids, was similar between scWAT and cMAT in both cohorts.

### Region-specific transcription factor expression

**Figure 7:**
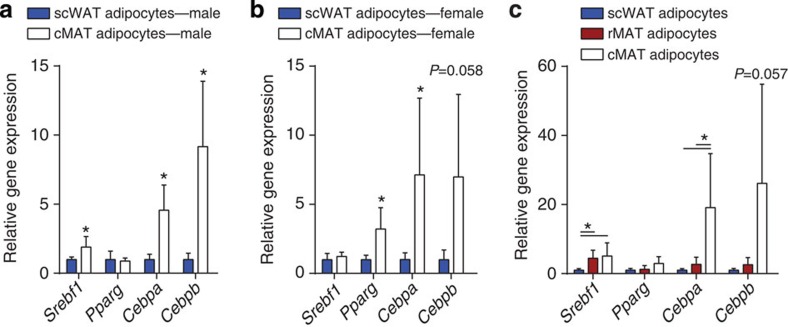
Gene expression of transcription factors in isolated adipocytes. Transcript expression in isolated adipocytes from subcutaneous WAT (scWAT), constitutive MAT (cMAT) and/or regulated MAT (rMAT) normalized to scWAT from (**a**) 16-week-old male Sprague–Dawley rats (biological replicate *N*=6 cMAT (two animals pooled per sample) and 12 scWAT), (**b**) 8-month-old female Sprague–Dawley rats (biological replicate *N*=3 cMAT (two animals pooled per sample) and 5 scWAT), and (**c**) 16-week-old male Sprague–Dawley rats (biological replicate *N*=5, four animals pooled per sample). Presented as mean±s.d. *(**a**,**b**) Two-tailed *t*-test, (**c**) one-way analysis of variance, *P*<0.05.

Differentiation of adipocytes from precursor cells is tightly regulated by a defined transcriptional cascade (see ref. 22 for review). The transcription factors CCAAT/enhancer-binding protein (C/EBP)-β and -δ are induced during early adipogenesis. These factors then activate expression of the essential adipogenic transcription factors peroxisome proliferator-activated receptor-γ and C/EBPα (ref. 23). Sterol regulatory element-binding protein-1 (encoded by *Srebf1*) serves as a transcriptional activator that is required for lipid homeostasis in mature adipocytes. Unexpectedly, in cMAT adipocytes, expression of both *Cebpa* and *Cebpb* was elevated relative to rMAT and/or scWAT adipocytes from male and female rats (Fig. 7a–c). Expression of *Srebf1* was elevated in cMAT and rMAT adipocytes of males, but not females. In contrast, *Pparg* was similar between cMAT/rMAT/WAT in males but increased in cMAT relative to scWAT in females. The similar or elevated expression of *Pparg* in MAT reinforces the notion that these cells are of the adipocyte lineage, but the selective elevation of *Cepba* and *Cebpb* in cMAT adipocytes suggests potential for alternative transcriptional regulation and function in this unique adipocyte population.

### Knockout of PTRF inhibits formation of rMAT adipocytes

Patients with CGL lose a majority of their peripheral WAT; however, magnetic resonance imaging (MRI) scans indicate that MAT is preserved in those with mutations in *CAV1* (CGL3) and *PTRF* (CGL4) (reviewed in ref. 2). Caveolin-1 (encoded by *CAV1*) is a key structural component of caveolae, 50–100 nm invaginations of the plasma membrane that account for up to 50% of the surface of peripheral white adipocytes^24^. *PTRF* encodes for cavin-1, a protein required for stabilization of caveolins and formation of caveolae^25^,^26^,^27^,^28^. Caveolae and their associated proteins coordinate many diverse signalling pathways and have been identified as key regulators of insulin sensitivity, lipid trafficking and adipocyte precursor differentiation^29^,^30^.

**Figure 8:**
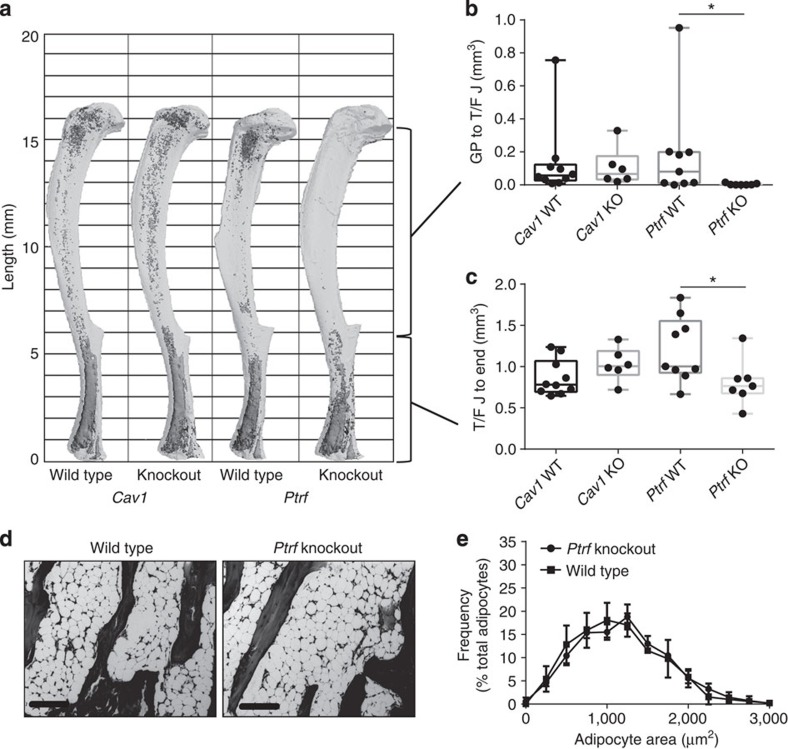
Differential loss of MAT in mice with knockout of *Cav1* or *Ptrf*. (**a**) Representative osmium-stained tibiae scanned with μCT based on data in **b** and **c**. Marrow fat in dark grey, decalcified bone overlaid in light grey. (**b**,**c**) Region-specific quantification of tibial MAT volume by μCT (biological replicate *N*=6–9 as indicated on the graph). Box plot centre line represents median, box extends from the 25th to 75th percentile, whiskers indicate range. (**d**) Representative histology of caudal vertebrae based on data in **e**, × 10 objective (scale bars, 200 μm). (**e**) Adipocyte size distribution of the caudal marrow adipocytes as measured by histology (biological replicate *N*=5). Histogram bin size 250. Presented as mean±s.d. *(**b**) Non-parametric Mann–Whitney test, (**c**) two-tailed *t*-Test, (**e**) two-way analysis of variance with Sidak’s multiple comparisons test, *P*<0.05. KO, knockout; WT, wild type.

To explore the preservation of MAT in CGL3 and CGL4, we quantified region-specific changes in MAT of adult male and female *Cav1* and *Ptrf* knockout mice at 16–17 weeks of age. The metabolic and peripheral adipose tissue phenotypes of these animals have been reported previously^26^,^31^,^32^,^33^. Consistent with the CGL3 human phenotype^34^, *Cav1* knockout mice did not lose MAT (Fig. 8a–c and Supplementary Fig. 4), despite a significant decrease in the amount of peripheral WAT (Supplementary Fig. 5A,B). As with MAT, trabecular bone at the proximal tibial metaphysis and cortical bone at the mid-diaphysis remained unchanged in the *Cav1* knockout animals (Supplementary Fig. 5C,D).

**Figure 9:**
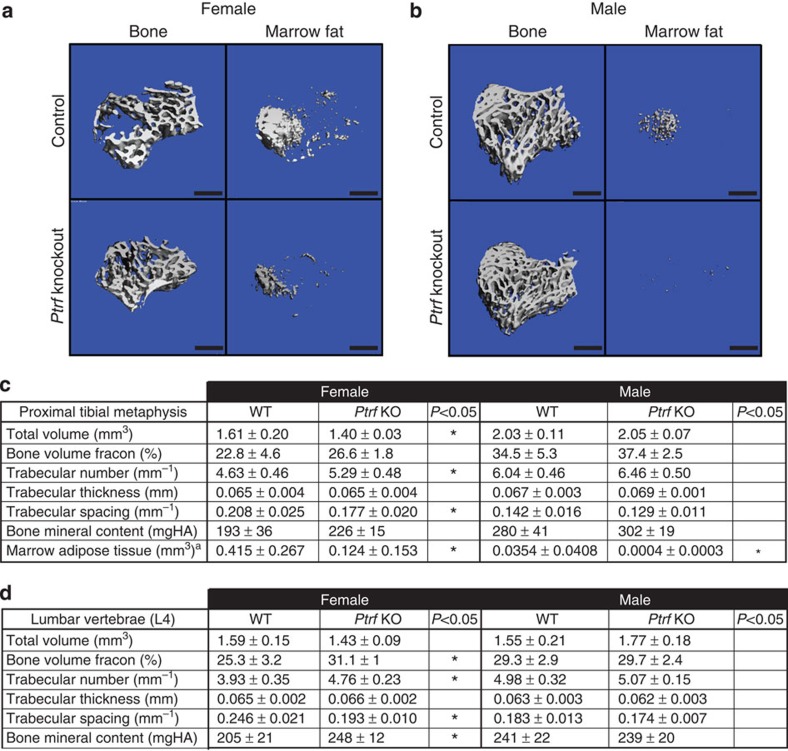
Trabecular morphology versus MAT in the tibia and L4 vertebrae of *Ptrf* KO mice. (**a**,**b**) Representative images of the proximal tibial metaphysis both before decalcification and after osmium staining based on data in c. Marrow fat is in white. Scale bars, 500 μm. (**c**) Quantification of trabecular parameters and MAT volume in the proximal tibial metaphysis (biological replicate *N*=5 (female KO), 6 (female WT), 7 (male KO) and 9 (male WT)). (**d**) Quantification of trabecular parameters in the vertebral body of L4 (biological replicate *N*=5 (WT) and 4 (KO)). KO, knockout; WT, wild type. All values represent mean±s.d. *Two-tailed *t*-test, *P*<0.05. ^a^Non-parametric Mann–Whitney test, *P*<0.05.

In addition to loss of WAT (Supplementary Fig. 6), in male mice knockout of *Ptrf* caused nearly complete loss of proximal tibial rMAT adipocytes with a relative preservation of cMAT in the distal tibia (Fig. 8a–c). Based on the three-dimensional reconstructions of the tibiae from *Ptrf* knockout animals, only the most distal portion of the MAT in the tibia was maintained while there was mixed preservation moving towards the tibia/fibula junction (Fig. 8a). This finding, similar to the lipidomic data in the rats, suggests a possible mixture of rMAT and cMAT adipocytes in the distal tibia. In contrast, the tail vertebrae of male *Ptrf* knockout mice remained completely filled with MAT (Fig. 8d), and these vertebral cMAT adipocytes were of the same size as those of wild-type animals (Fig. 8e). Except for a 4.6% increase in cortical bone mineral content, trabecular and cortical parameters did not differ between the *Ptrf* knockout males and their wild-type counterparts (Fig. 9c and Supplementary Fig. 6).

Similar decreases in rMAT and WAT were observed in the female *Ptrf* knockout mice (Fig. 9a and Supplementary Figs 4 and 6). Unlike males, the female *Ptrf* knockout mice had a significant 14.3% increase in trabecular number and corresponding 8.5% decrease in trabecular spacing (Fig. 9c). In addition, relative to males, both control and *Ptrf* knockout females had increased MAT volume in the proximal tibia (Fig. 9) that was inversely correlated with trabecular number at the proximal tibial metaphysis (*P*=0.021). Interestingly, the trabecular bone phenotype of the females was even more striking in the L4 vertebral body, with a 21.1% increase in trabecular number, 21.5% decrease in spacing, 22.9% increase in bone volume fraction and 21.0% increase in bone mineral content (Fig. 9d). Consistent with previous reports^10^, we did not observe MAT adipocytes in the lumbar vertebrae in either the wild-type or knockout females. As with the tibia, the trabecular phenotype of the vertebrae was unaffected by *Ptrf* knockout in males.

## Discussion

**Figure 10:**
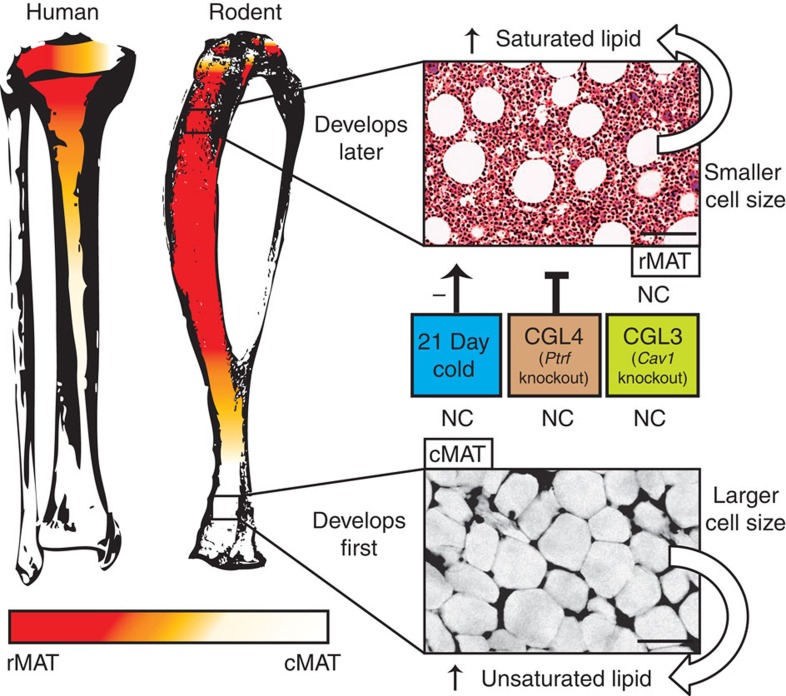
rMAT versus cMAT summary. In the human and mouse tibia, cMAT is present in the distal portion of the bone. The marrow shifts to red towards the proximal tibia, occurring near the tibia/fibula junction in the rodent and in the proximal tibial metaphysis or femur in the human. The red marrow contains rMAT adipocytes. In some cases, especially in larger species such as humans, the histological patterns that correspond to rMAT and cMAT adipocytes may be present in the same region. The bones have been shaded in orange to indicate this possibility. cMAT is the first to develop and histologically appears as sheets of confluent adipocytes that are relatively devoid of haematopoiesis. Isolated cMAT adipocytes form shortly after birth, have an increased proportion of unsaturated fatty acids and are larger in size. rMAT develops throughout life and is histologically defined as single cells interspersed with areas of active haematopoiesis. Isolated rMAT adipocytes have a lipid saturation profile that is similar to WAT adipocytes and are more saturated, and smaller in size, than cMAT. These cells are negatively regulated by 21-day cold exposure. They also fail to form in mice with genetic knockout of *Ptrf*, but not *Cav1*. NC, no change. Scale bar, 50 μm.

Our results demonstrate that there are region-specific differences in development, regulation, adipocyte size, lipid composition, gene expression and genetic determinants of marrow adipocytes that have implications for understanding the marrow niche and its relationship to skeletal and whole-body metabolism. Localization of osmium-stained adipocytes in three dimensions demonstrated that MAT in mice develops asymmetrically from distal to proximal. A similar pattern of early development occurs in vertebrae species including rats, rabbits and humans. However, the absolute rate of formation decreases with increasing lifespan/size of the animal. For example, the ‘adult’ distribution of MAT in humans occurs around age 25 years, in rabbits by 6 months and in mice as early as 8 weeks—likely with some relationship to sexual maturation^12^,^13^,^35^,^36^. The amount of MAT that forms during this phase also varies between species; larger animals have more MAT that extends farther into the skeleton than smaller animals (humans > rabbits > rats > mice). In addition, we found that MAT forms in two distinct temporal waves that are spatially separated in mice and correspond histologically to rMAT in red marrow and cMAT in yellow marrow (Fig. 10). Conversely, MAT loss with cold exposure is the opposite of development—the last to form is the first to go. The cMAT in the distal tibia and tail, in particular, is highly resistant to dissolution. While the microenvironment likely plays a major role in these site-specific responses, our lipidomic and gene expression data identify cell autonomous differences between the rMAT and cMAT adipocytes that might also contribute to their distinct behaviours.

Tavassoli^11^, in 1976, demonstrated the presence of two different types of adipocytes in rabbit bone marrow—those that stain with performic acid Schiff (PFAS) and those that do not. The stain reaction is thought to rely on oxidation of the ethylenic linkages in unsaturated fats to aldehyde and processing with Schiff’s reagent to generate a red/purple colour, although this mechanism is controversial^37^. Inspired by Tavassoli’s^11^ PFAS stain, we found that rMAT and cMAT have distinct lipidomic profiles (Fig. 5 and Supplementary Data 1). In addition, despite the histological similarities between WAT and cMAT—the lipid composition of WAT more closely mirrors that of rMAT, suggesting that lipid metabolism in WAT and rMAT adipocytes may be similar. Coordinate regulation by cold exposure and similarities in *Pparg*, *Cebpa* and *Cebpb* gene expression between rMAT and WAT further support this hypothesis. Of note, the increase in cMAT unsaturation is actually the opposite of what we expected based on the proposed mechanism of the PFAS stain. This is likely due to the historic debate surrounding the stain, which in one paper from 1970 was characterized as ‘useless in lipid histochemistry’^37^. Regardless, it led us to uncover a highly conserved difference between rMAT and cMAT in rats that, based on indirect evaluation with ^1^H-MRS, appears to extend to human MAT.

These findings have implications for diseases including osteoporosis, which has been associated with a decrease in MAT unsaturation^38^. A shift in marrow fat composition to higher levels of saturated lipid has also been correlated with fragility fractures in postmenopausal women^39^. For example, palmitate is lipotoxic to osteoblasts and impairs mineralization^40^. As a proportion of total lipid, palmitate is enriched in rMAT relative to cMAT. In contrast, palmitoleate, which is enriched in cMAT, has been identified as a secreted adipose tissue-derived lipid hormone with the capacity to stimulate muscle insulin action and suppress hepatosteatosis^41^. Our current analysis does not discriminate between lipid types (for example, triacylglycerols versus phospholipids); hence, future work is needed to examine subcellular localization and secretion of fatty acids, in addition to other mediators, by rMAT and cMAT, and to quantify their impact on local and distant tissues.

In the introduction, we highlighted the unresolved controversy that exists regarding the relationship between MAT and bone. It is notable that the reports that correlate MAT accumulation with low bone mineral density, decreased bone formation and bone loss generally analyse rMAT-enriched sites, including the proximal femur, hip and lumbar spine (reviewed in ref. 2). In contrast, studies demonstrating resistance to bone loss at sites of high MAT are all based on cMAT-enriched areas including the distal tibia and tail vertebrae^7^,^8^,^9^. In this manuscript, we explored changes in trabecular and cortical architecture and compared our findings with MAT volume and its three-dimensional distribution. During development in B6 and C3H mice, we observed polarization of rMAT towards the medial marrow space in the proximal tibia and to the medial/posterior endocortical surface at the mid-tibia (Fig. 2). MAT accumulation from 12 to 56 weeks of age correlated negatively with trabecular number and positively with trabecular thickness in both strains. Conversely, extensive loss of MAT in the proximal tibia in mice undergoing 21-day cold exposure failed to uniformly impact trabecular or cortical parameters (Supplementary Fig. 1). In our genetic models, knockout of *Cav1* left both MAT and bone unchanged. In contrast, developmental inhibition of rMAT accumulation in the female *Ptrf* knockout mice was correlated with increased trabecular number in the proximal tibia. With this phenotype, it is tempting to conclude that rMAT loss is necessary for trabecular gain; however, in this same model, the increase in trabecular number was actually more pronounced in the lumbar vertebrae—a skeletal site in the mouse that has little to no MAT.

What then can we conclude about the relationship between MAT and bone? It is certainly of note that developmental polarization of MAT along the medial and posterior surfaces of the cortical bone implies that rMAT may be related to cortical drift patterns during development. Similarly, logic dictates that accumulation of MAT in the proximal tibia must occur at the expense of either haematopoiesis or bone, since the size of the space within the skeleton is finite. It would not be unreasonable to subsequently assume that these components have an inherent ability to regulate one another. What we truly need, however, are animal models in which we can specifically regulate rMAT and cMAT *in vivo*. Identification of *Ptrf* knockout as a selective mediator of rMAT loss (Fig. 8) is one step towards generation of a genetic model of rMAT ablation. Future quantification of MAT, expanded beyond conventional methods to include both rMAT and cMAT, in currently available genetic models will undoubtedly reveal additional targets.

Although depletion of MAT has been proposed as a strategy to combat osteoporosis^42^, the function of rMAT and cMAT must be clarified before removal of MAT populations. The highly defined accumulation of cMAT early in vertebrate development and its robust resistance to dissolution implies an important function for this adipose depot—one that may go beyond the skeleton^1^. In contrast, rMAT adipocytes are more closely situated in areas of high bone turnover and are better positioned to actively influence haematopoiesis and/or skeletal remodelling. Although our data provide a working definition of rMAT and cMAT, and highlight the need to explore these cells in more detail (Fig. 10), there are many questions that remain. Our ability to definitively address fundamental differences in marrow adipocytes and their role locally in the skeletal microenvironment, or systemically, as a component of whole-body metabolism, depends on future development of targeted animal models and continued clinical investigation.

## Methods

### Rodents

Where they were utilized, animal procedures were approved by the animal use and care committees at the University of Michigan, Maine Medical Center Research Institute and/or Boston University. Animals were housed at 22 °C on a 12-h light/dark cycle unless otherwise indicated.

*Development*. Male C57BL/6J (Jackson Labs, stock: 000664) and C3H/HeJ (Jackson Labs, stock: 000659) mice were euthanized at 1, 4, 12 or 56 weeks of age and tissues were collected for analysis. The 12- and 56-week-old C3H animals are the same as the control groups for the C3H cold exposure experiment outlined below.

*Cold exposure*. At 8 or 52 weeks of age, 10 male C3H/HeJ (Jackson Labs, stock: 000659) mice were placed individually into pre-cooled cages with bedding, food and water in a room held at 18 °C. Littermate control mice (*n*=10 per strain) were held in identical conditions at room temperature (∼22 °C). After 1 week at 18 °C, the cold room was adjusted to 4 °C and maintained at this temperature for an additional 3 weeks. Control mice were held at 22 °C for a total of 4 weeks (concurrently). Rectal core body temperature of control and cold-exposed mice was monitored daily using a Type T thermocouple rectal probe (RET-3, Physitemp Instruments, Inc., Clifton, NJ, USA) with a MicroTherma 2T hand-held thermometer (ThermoWorks, Inc., Lindon, UT; cat. no. THS-227-193). After 4 weeks, all mice were killed and tissues were collected for analysis. Given the length of the intervention (21 days), we pre-scanned the non-decalcified tibia bones to calculate the marrow volume. After decalcification and osmium stain, the bones were re-scanned and the MAT volume was normalized to marrow volume in each region of interest to correct for any changes in the size of the tibiae between groups.

*Cav1 and Ptrf knockout mice*. *Cav1* and *Ptrf* knockout mice were generated previously^26^,^31^. Homozygous Cav^tm1Mls/J^ mice with knockout of *Cav1* on a mixed background (Jackson Labs, stock: 004585) were crossed with B6129SF2/J controls (Jackson Labs, stock: 101045). The resulting *Cav1*^+/−^ heterozygotes were crossed and the male homozygous offspring were euthanized for analysis at 16 weeks of age. The homozygous male and female *Ptrf*^−/−^ and their wild-type control littermates were generated from breeding *Ptrf*^+/−^ heterozygotes and used for the present study at 16–17 weeks of age. The *Ptrf*^−/−^ mice had previously been backcrossed to C57BL/6J mice for at least nine generations.

### Marrow fat quantification by osmium staining and CT

Mouse bones were stained with osmium tetroxide for analysis of marrow fat, with slight modification from ref. 17, as follows. Bones were fixed in 1.5 or 2.0 ml microtubes for 24–48 h in 10% neutral-buffered formalin (VWR, Radnor, PA; cat. no. 16004-128), washed with water and decalcified in 14% EDTA, pH 7.4, for 14 days. After washing again with water, 600 μl Sorensen’s phosphate buffer (pH 7.4) was added to one bone (femur or tibia) in a 1.5-ml microtube. (Note: all subsequent steps must be performed in the fume hood.) Four per cent osmium tetroxide (200 μl) solution (Electron Microscopy Services, Hatfield, PA; cat.no. 19170) was added to each tube to make a 1% solution. Bones were stained in the fume hood 48 h at room temperature. Osmium solution was carefully removed to a small liquid waste container that had been filled with corn oil to ∼25% of the volume. Any used pipet tips were ‘rinsed’ of active osmium tetroxide by pipeting corn oil. All tips and tubes were discarded as osmium solid waste. Bones were washed, in the same tube, by incubating in 1 ml of Sorensen’s buffer for 3 h at room temperature. This was repeated twice and the last wash was left in the hood overnight. This waste was disposed of as indicated above. Stained bones were then moved to a fresh set of 1.5 ml microtubes containing 1 ml Sorensen’s buffer each. The used tubes were discarded as solid osmium waste. At this point, the bones and tubes were removed from the fume hood and used for CT.

*MicroCT*. Specimens were embedded in 1% agarose and placed in a 19-mm diameter tube. The length of the bone was scanned using a μCT system (μCT100 Scanco Medical, Bassersdorf, Switzerland). Scan settings are as follows: voxel size 12 μm (all except Fig. 1a, 1 week bones at 10 μm), medium resolution, 70 kVp, 114 μA, 0.5 mm AL filter and integration time 500 ms. Density measurements were calibrated to the manufacturer’s hydroxyapatite phantom. Analysis was performed using the manufacturer’s evaluation software and a threshold of 400 for MAT.

*NanoCT*. Samples were scanned at 2 μm voxel size, 90 kV, 90 μA and 1,500 ms exposure time with a total scan time of 73 min on a nanotom-s (phoenix|X-ray, GE Measurement & Control; Wunstorf, Germany). DICOM image files were opened in ImageJ^43^ for size analysis of individual adipocytes in two dimensions using a ‘virtual histology’ approach (Supplementary Fig. 1). The area of 300–500 adipocytes was measured per sample^44^. A bin size of 250 was used to generate a size distribution histogram for each adipocyte type. The average adipocyte volume was estimated based on the average adipocyte area and compared with the total MAT volume (as determined by μCT) to determine the number of adipocytes in a region of interest.

### Histology

Samples were fixed in 10% neutral-buffered formalin and decalcified in 14% EDTA, pH 7.4, before paraffin embedding and haematoxylin and eosin stain. Where indicated, osmium-stained bones (prepared as detailed above) were submitted and processed in the same way.

### Human marrow unsaturation

This study was approved by the Partners Healthcare Institutional Review Board and complied with Health Insurance Portability and Accountability Act guidelines. Written informed consent was obtained from all subjects after the nature of the procedure had been fully explained. We studied five women (mean age: 33±10 years) with a mean body mass index (BMI) of 24.8±10 kg m^2^. All subjects underwent proton MRS (^1^H-MRS) of the proximal femoral metaphysis, the mid-femoral and tibial diaphyses, and the distal tibial metaphysis to determine MAT content and composition using a 3.0-T MR imaging system (Siemens Trio, Siemens Medical Systems, Erlangen, Germany). Single-voxel ^1^H-MRS data were acquired using point-resolved spatially localized spectroscopy pulse sequence without water suppression as previously described^18^. Coefficient of variation for bone marrow fat quantification was 5%. Fitting of all ^1^H-MRS data was performed using LCModel (version 6.3-0K) as previously described^18^. A customized fitting algorithm for bone marrow analysis provided estimates for total marrow lipid content (lipid peaks at 0.9, 1.3, 1.6, 2.0 and 5.3 p.p.m. combined). Unsaturation index was determined by obtaining a ratio between the olefinic resonance at 5.3 p.p.m., an estimate of fatty-acid unsaturation bonds, and total lipid content.

### Adipocyte isolation for lipidomics

Adipocytes were isolated from rat WAT and MAT using a modified collagenase digestion protocol as described below^19^. Older female rats were obtained from the University of Michigan rat recycling programme and including 1-year-old female high-capacity runner rats (*N*=3; ref. 20) and ∼8-month-old female Sprague–Dawley rats (*N*=5). Sixteen-week-old male Sprague–Dawley rats were obtained from Charles River Laboratories (strain code: 400). Rats were anaesthetized with isofluorane in a drop jar and euthanized by decapitation. The processing for each sample type is outlined in detail below. All protocols were performed simultaneously and the adipocytes from each tissue underwent methanol–choloroform extraction for total lipid at the same time (±5 min).

*White adipose tissue*. Adipose tissues were removed and placed in warm Krebs-Ringer HEPES (KRH) buffer, pH 7.4 (10 mM HEPES, 120 mM NaCl, 1.2 mM KH_2_PO_4_, 1.2 mM MgSO_4_, 2.5 mM CaCl_2_, 15 mM NaHCO_3_, 4.8 mM KCl, 1.0 g l^-1^ D-glucose and 500 nmol adenosine), that had been pre-equlibrated overnight in an incubator at 37 °C, 5% CO_2_ and re-pHed to 7.4. Washed adipose tissue pieces totalling ∼1 g were minced in 10 ml KRH containing 1 mg ml^-1^ collagenase type I (Worthington Biochemical Corp., Lakewood, NJ; cat. no. 4197) and 3% fatty-acid-free BSA (Calbiochem; cat. no. 126575) in a 50-ml conical tube and placed in a shaking water bath at 100 r.p.m., 37 °C for 45–60 min. Digested tissue was pulled gently through a 10-ml polypropylene Luer-lock syringe (no needle) three times to complete disruption and then filtered through a 100-μm cell strainer (Fisherbrand, Pittsburgh, PA; cat. no. 22363549) into a fresh 50-ml polypropylene conical tube.

*Tibial cMAT*. Both tibiae were removed and cleaned of muscle and tendon using gauze. A rotary power tool (Dremel, Robert Bosch Tool Co, Addison, IL) with a Dremel 545 Diamond cutting wheel was used to horizontally bisect the tibia at the base of the tibia/fibula junction. The distal portion was inverted into a 1.5-ml polypropylene microtube containing a hollow spacer and centrifuged at 3,000*g* to extrude the marrow. The bone was removed and discarded, and the distal tibial marrow was then processed in the same manner as the WAT, described above.

*Femur/tibia rMAT*. Both femurs were isolated and cleaned, and the ends were removed with the rotary tool to expose the marrow cavity. The femurs and the proximal tibiae were inverted into 1.5 ml microtubes and centrifuged at 3,000*g* to separate the marrow. The bones were discarded. Gentle pipetting was used to combine and resuspend the proximal marrow in KRH containing 1 mg ml^-1^ collagenase and 3% BSA in a 50-ml conical tube. The suspension was then incubated in a shaking water bath at 100 r.p.m., 37 °C for 15–20 min to liberate the rMAT adipocytes.

*Vertebral cMAT*. The most proximal 10 tail vertebrae were separated and some of the surrounding muscle and tendon were removed with gauze. The vertebrae were added to a 50-ml conical tube with 2 × the volume of KRH+1 mg ml^-1^ collagenase and 3% BSA. The tube was then incubated in a shaking water bath at 100 r.p.m., 37 °C for 20 min, with vigorous shaking by hand every 5 min to help dislodge remaining tissue on the outside of the vertebrae. After 20 min, the vertebrae solution was poured into a 10-cm dish. The vertebrae were quickly cleaned with gauze to remove any remaining soft tissue. Each vertebrae was then bisected longitudinally with a diagonal cutter and put into a fresh 50-ml conical tube containing 2 × the volume of KRH/collagenase/BSA solution. The bisected vertebrae were incubated in a shaking water bath at 100 r.p.m., 37 °C for an additional 20–30 min to liberate the cMAT adipocytes.

*Vertebral rMAT*. Eight lumbar vertebrae were isolated and cleaned with gauze. The processing then continued as described for the vertebral cMAT above.

Final processing for all adipocyte types. After filtration, the conical tubes were centrifuged at 400*g* for 1 min to pellet the stromal vascular fraction and float the adipocytes. The pellet and the majority of the infranatant was carefully removed with a glass pipet and suction bulb. A plastic 1,000 ml pipet tip was used to resuspend the adipocytes and transfer 300 μl of liquid containing 0.1–1.0 mg of cells to a 24-well plate size transwell insert with 8 μm pores (Corning Inc., Corning, NY; cat. no. 3422). Approximately 90% of the liquid was removed by pressing the transwell membrane on a piece of dry paper towel. The cells in the insert were then washed twice in this manner with fresh KRH (no collagenase, no BSA). After the final wash and liquid depletion, the cells in the insert were collected in 300 μl of water and transferred immediately to a borosilicate glass tube for lipid extraction as described below.

### Lipidomic analyses of rat adipocytes

*Lipid extraction*. Lipids from the adipocyte samples were extracted following essentially the Bligh and Dyer^45^ method of solvent partition. A typical extraction procedure consists of suspending the cells in a borosilicate glass tube in 0.3 ml of water followed by adding 1.125 ml of a mixture of chloroform–methanol (1:2). The mixture was then vortexed to disrupt the cells. The samples were further treated with 0.375 ml each of chloroform and NaCl (0.9%) solution followed by vortexing and centrifugation at 4 °C, 6,500*g*, for 7 min. The lower organic (chloroform) layer containing the total lipids was separated out and saved at −20 °C for further use.

*Preparation of methyl ester with boron trifluoride–methanol and purification*. The fatty-acid components of the lipids were derivatized into their methyl esters via trans-esterification with boron trifluoride–methanol^46^ after slight modification as follows. The solvents of the above lipid extract were removed under nitrogen. To the dry residue, 2 ml of boron trifluoride–methanol (14% solution) and 10 μl of 4 mM heptadecanoic acid (C_17_) as an internal standard were added, and the tubes containing the mixture were closed under nitrogen and incubated at 68 °C for 3–3.5 h. The methyl esters were extracted by adding 2 ml of hexane and 1 ml of water, mixing followed by centrifugation. The hexane layer containing methyl esters was transferred into a separate tube. The solvent was then removed under nitrogen, the methyl esters were re-dissolved in to a small volume of hexane and purified by thin-layer chromatography (TLC) using *n*-hexane-diethyl ether–acetic acid (50:50:2, v/v) as the developing solvents^47^, applying authentic standard fatty-acid methyl ester (FAME) side by side on the TLC plate. After development, the plates were dried and sprayed with Premulin^48^. The products were identified with respect to the retention flow of the standard (retention flow=0.67). The methyl esters were extracted from the TLC powder with diethyl ether, the volumes were concentrated under nitrogen, re-dissolved in 100 μl of hexane and the fatty-acid compositions of the lipids were analysed by GC as follows.

*GC of FAME*. Analysis of FAMEs was performed with 1 μl of sample injection, by GC on an Agilent GC machine, model 6890N equipped with flame ionization detector, an auto sampler and a Chemstation software for data analysis. The GC column used was Agilent HP 88, 30 m, 0.25 mm I.D. and film thickness 0.20 μm. Hydrogen was used as a carrier gas as well as for flame ionization detector and nitrogen was used as a makeup gas. Analyses were carried out with a temperature programming of 125–220 °C. The fatty-acid components in unknown samples were identified with respect to the retention times of standard methyl ester mixtures run side by side. The fatty-acid components were quantified with respect to the known amount of C_17_ internal standard added and the calibration ratio derived from each fatty acid of a standard methyl esters mixture and the methyl heptadecanoate (C_17_) internal standard.

### Adipocyte isolation for quantitative PCR

Adipocytes were isolated from two cohorts of animals, 16-week-old male Sprague–Dawley rats and ∼8-month-old female Sprague–Dawley rats as described above. Adipocytes, including rMAT from the proximal tibia and femur, were then isolated from a third cohort of 16-week–old male Sprague–Dawley rats by adding 50 U ml^-1^ heparin to the collagenase solution. Adipocyte preparations were lysed and processed using Stat60 reagent (Amsbio, Cambridge, MA, USA) to isolate total RNA. Pelleted RNA was resuspended in water and 100 μg of total RNA was reverse-transcribed to cDNA using TaqMan RT reagents (Applied Biosystems, Carlsbad, CA, USA). Quantitative PCR was performed using qPCRBIO SyGreen 2 × mix, Hi-Rox, on an Applied Biosystems real-time PCR detection system (Applied Biosystems). Gene expression was calculated based on a cDNA standard curve within each plate and normalized to the expression of TATA-binding protein (TBP) messenger RNA. Primer sequences are presented in Supplementary Table 1.

### Statistics

Graphpad Prism software was used to perform statistical tests. Tests including a two-tailed, homoscedastic *t*-test, a non-parametric Mann–Whitney test, two-way analysis of variance with Sidak’s multiple comparisons test and one-way analysis of variance with Tukey’s multiple comparisons test were applied as indicated in the figure legends. Principal components analysis was performed using MetaboAnalyst^21^. When possible, sample size was determined based on the effect size of preliminary data, and data analysis was performed by an investigator that was blinded to the sample groups.

## Additional information

**How to cite this article:** Scheller, E. L. *et al*. Region-specific variation in the properties of skeletal adipocytes reveals regulated and constitutive marrow adipose tissues. *Nat. Commun*. 6:7808 doi: 10.1038/ncomms8808 (2015).

## Supplementary Material

Supplementary Figures and Table Supplementary Figures 1-6 and Supplementary Tables 1

Supplementary Data 1 Total lipid was extracted from isolated adipocytes with methanol-choloroform and then used gas chromatography for lipidomic analysis of esterified fatty acids. Column headings are in the following format ExN-YZ where N is the experiment number, Y is the rat number, and Z is the source tissue type. (Experiment #1, Ex1) one-year-old female high-capacity-runner rats; (Experiment #2, Ex2) 16-week-old male Sprague Dawley rats; and (Experiment #3, Ex3) 8-month-old female Sprague Dawley rats. Sources of adipocytes include pooled femur and proximal tibia marrow (FpT), lumbar vertebral marrow (LV), caudal vertebrae (CV), distal tibial marrow (dT), inguinal white adipose tissue (i), gonadal white adipose tissue (g), and perirenal white adipose tissue (pr). Fatty acid subtypes for each sample are expressed as a percent of the total lipid.
