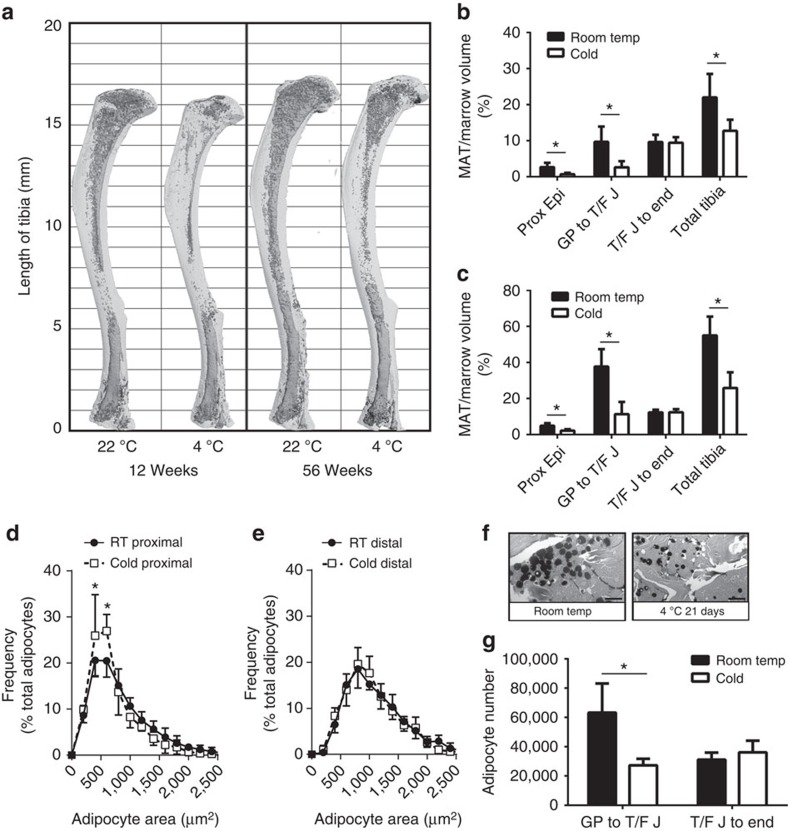# Corrigendum: Region-specific variation in the properties of skeletal adipocytes reveals regulated and constitutive marrow adipose tissues

**DOI:** 10.1038/ncomms13775

**Published:** 2016-12-08

**Authors:** Erica L. Scheller, Casey R. Doucette, Brian S. Learman, William P. Cawthorn, Shaima Khandaker, Benjamin Schell, Brent Wu, Shi-Ying Ding, Miriam A. Bredella, Pouneh K. Fazeli, Basma Khoury, Karl J. Jepsen, Paul F. Pilch, Anne Klibanski, Clifford J. Rosen, Ormond A. MacDougald

Nature Communications 6: Article number: 7808; DOI: 10.1038/ncomms8808 (2015); Published: 08 06 2015; Updated: 12 08 2016

In Figure 3 of this Article, marrow adipose tissue volumes (in panels b and c) and adipocyte numbers (in panel g) were calculated incorrectly. In Fig. 3b,c, regional MAT volumes in the ‘GP to T/F J' group were normalized incorrectly to both regional marrow volume and total marrow volume instead of normalizing to total marrow volume only. As a result, the correct MAT/marrow percentages in the ‘GP to T/F J' group are higher than those published. Furthermore, in Fig. 3g, the formula used for calculating adipocyte volumes was [(4/3) × pi × (radius)^2^], and this should have read [(4/3) × pi × (radius)^3^]. As a result, the correct absolute adipocyte numbers are higher than those published. However, relative differences between all groups in this panel remain the same. None of these alterations change the original conclusions. The correct version of the figure appears below as Fig. 1.

## Figures and Tables

**Figure 1:**