# Supplementary material for: Region-specific variation in the properties of skeletal adipocytes reveals regulated and constitutive marrow adipose tissues
Source: Nat Commun. 2015 Aug 6;6:7808. doi: 10.1038/ncomms8808 (PMC4530473; doi:10.1038/ncomms8808)
Supplement: Supplementary Figures and Table — Supplementary Figures 1-6 and Supplementary Tables 1 [file ncomms8808-s1.pdf]

SUPPLEMENTARY FIGURE 1

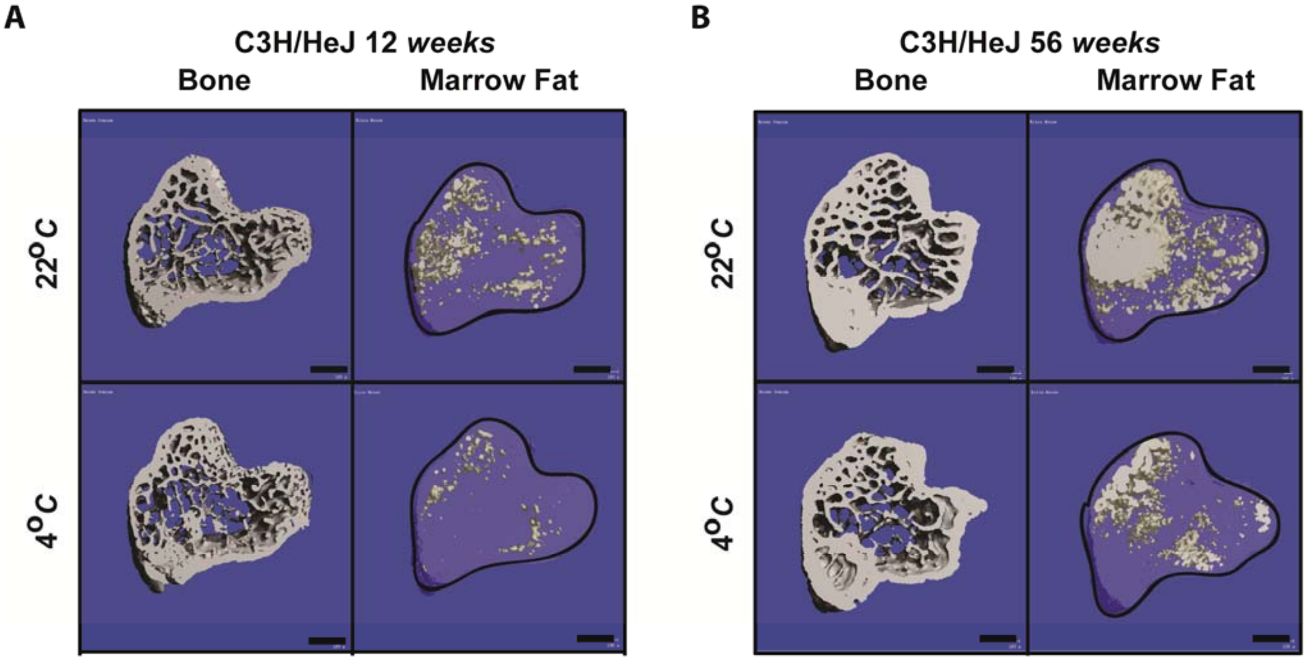

C

	12 *weeks*			56 *weeks*		
**Proximal Tibial Metaphysis**	**Room Temp**	**4°C**	**P < 0.05**	**Room Temp**	**4°C**	**P < 0.05**
Total Volume (*mm*^{3})	1.39 +/- 0.08	1.43 +/- 0.08		1.43 +/- 0.12	1.49 +/- 0.04	
Bone Volume Fraction (*%*)	22.5 +/- 3.4	22.4 +/- 3.4		28.3 +/- 4.5	28.8 +/- 2.1	
Trabecular Number (*mm*$^{-1}$)	5.25 +/- 0.36	5.25 +/- 0.38		4.45 +/- 0.59	4.70 +/- 0.42	
Trabecular Thickness (*mm*)	0.062 +/- 0.005	0.062 +/- 0.004		0.073 +/- 0.003	0.072 +/- 0.007	
Trabecular Spacing (*mm*$^{-1}$)	0.174 +/- 0.014	0.176 +/- 0.015		0.218 +/- 0.034	0.204 +/- 0.022	
Bone Mineral Content (*mgHA*)	196 +/- 28	197 +/- 26		254 +/- 33	258 +/- 24	
Marrow Adipose Tissue (*mm*^{3})	0.117 +/- 0.059	0.022 +/- 0.020	*	0.752 +/- 0.122	0.263 +/- 0.130	*

D

		12 *weeks*			56 *weeks*		
	**Tibia Mid-Cortical Morphology**	**Room Temp**	**4°C**	**P < 0.05**	**Room Temp**	**4°C**	**P < 0.05**
**3D**	Total Volume (*mm*^{3})	0.394 +/- 0.013	0.399 +/- 0.021		0.478 +/- 0.025	0.494 +/- 0.017	
	Relative Cortical Volume (*%*)	72.1 +/- 1.1	70.7 +/- 1.6	*	72.6 +/- 1.3	72.9 +/- 1.8	
	Bone Mineral Content (*mgHA*)	890 +/- 25	875 +/- 27		967 +/- 24	969 +/- 27	
**2D**	Total Area (*mm*^{2})	1.128 +/- 0.036	1.147 +/- 0.062		1.373 +/- 0.073	1.418 +/- 0.048	
	Cortical Bone Area (*mm*^{2})	0.813 +/- 0.027	0.811 +/- 0.040		0.996 +/- 0.058	1.033 +/- 0.045	
	Cortical Thickness (*mm*)	0.279 +/- 0.011	0.271 +/- 0.012		0.312 +/- 0.012	0.317 +/- 0.016	

Supplementary Figure 1. Trabecular and cortical analyses with cold exposure. (A,B) Representative images of the proximal tibial metaphysis both prior to decalcification and after osmium staining of the data presented in (C). Marrow fat is in white. Scale bar = 500 µm. **(C)** Quantification of trabecular parameters and MAT volume in the proximal tibial metaphysis (biological replicate N = 9 (C3H 12-week-old), and 11 (B6 12-week-old, 56-week-old)). **(D)** Quantification of cortical parameters (biological replicate N = 9 (C3H 12-week-old), and 11 (B6 12-week-old, 56-week-old)). All values represent mean ± standard deviation. *Two-tailed *t*-Test, p<0.05.

SUPPLEMENTARY FIGURE 2

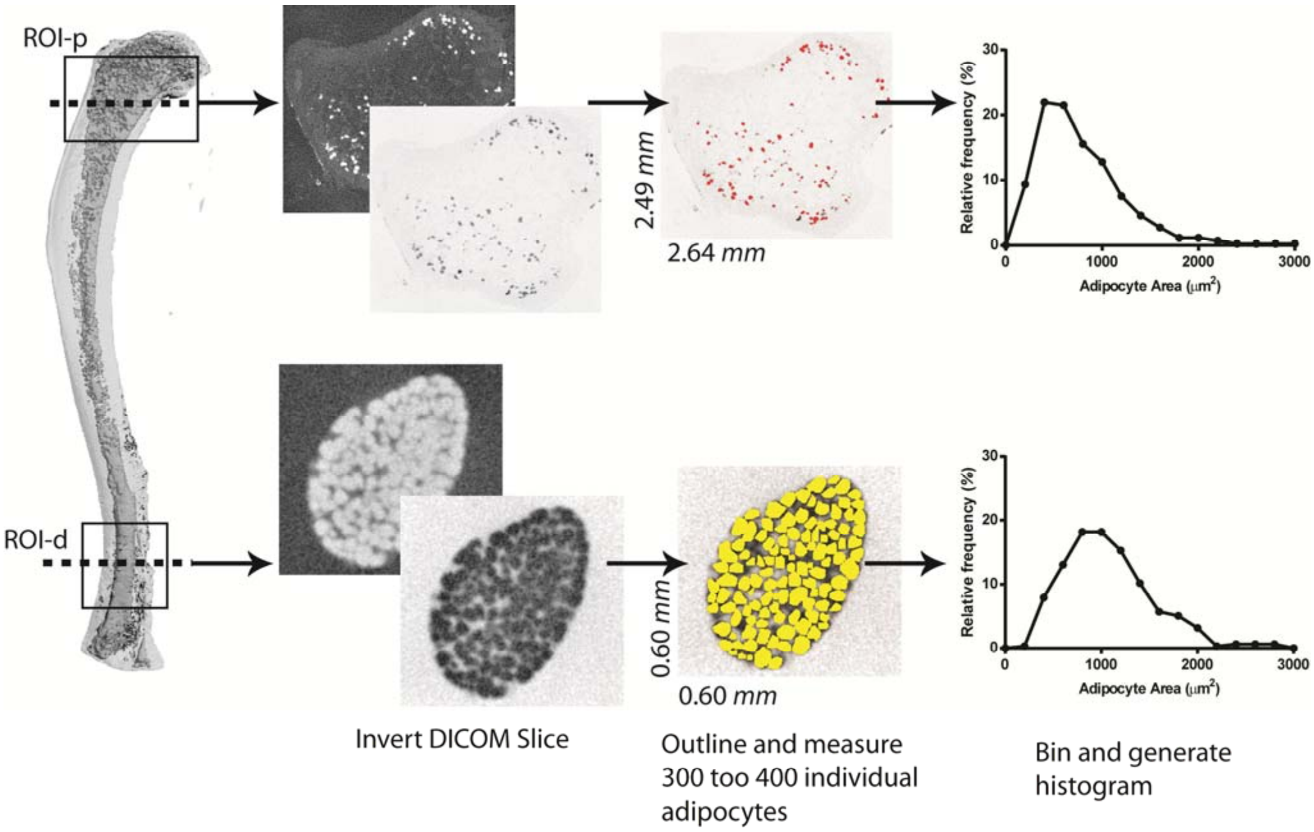

Supplementary Figure 2. NanoCT for quantification of adipocyte size. To quantify tibial adipocyte size using nano-computed-tomography, two regions of interest were scanned at 2 µm resolution. To measure rMAT adipocytes we scanned at the proximal metaphysis (ROI-p). To measure cMAT adipocytes we scanned the mid-portion of the distal tibia (ROI-d). Three to five DICOM images, each at least 100 µm apart were selected for analysis from each sample. The DICOM slice was opened in ImageJ and inverted to make visualization of the adipocytes easier. Most of the proximal adipocytes could be selected for analysis by the program, but the distal adipocytes were drawn by hand. With either method, each adipocyte outline was checked against the un-rendered image to confirm its accuracy. The area within each outline was then measured. After 300 to 400 adipocytes had been identified for analysis the frequency of each adipocyte area, with a bin size as indicated in the figures, was calculated. This size distribution was then represented as a histogram.

SUPPLEMENTARY FIGURE 3

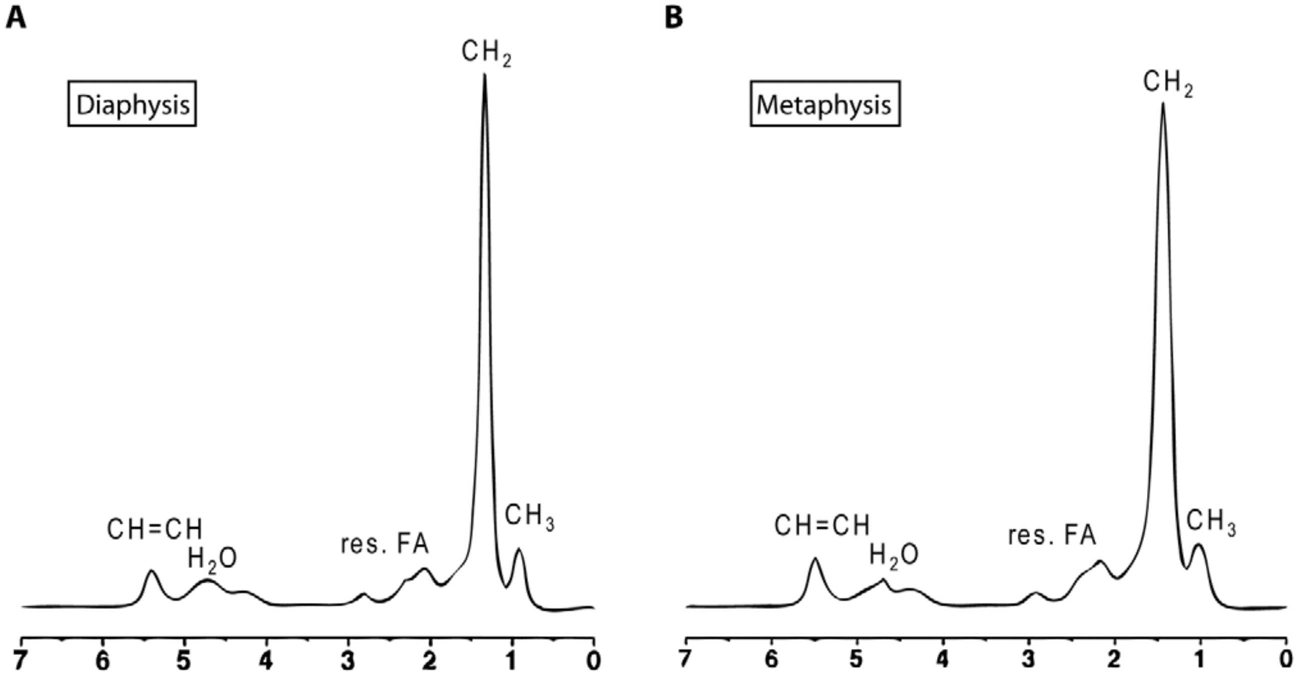

Supplementary Figure 3. Human ^{1}H-MRS. Spectra at the **(A)** mid-tibial diaphysis and **(B)** distal tibial metaphysis demonstrate olefinic protons at 5.3 ppm (-CH=CH-), an estimate of fatty acid (FA) unsaturated bonds, methylene protons at 1.3 ppm [(-CH2-)n], methyl protons at 0.9 ppm (-CH3), and residual (res.) FA including allylic and diallylic methylene protons. Total marrow lipid content was determined by combining all lipid peaks. Unsaturation index (UI) was determined by obtaining a ratio between the olefinic resonance at 5.3 ppm and total lipid content. Lipid resonances were scaled to unsuppressed water peak (H2O) at 4.7 ppm.

SUPPLEMENTARY FIGURE 4

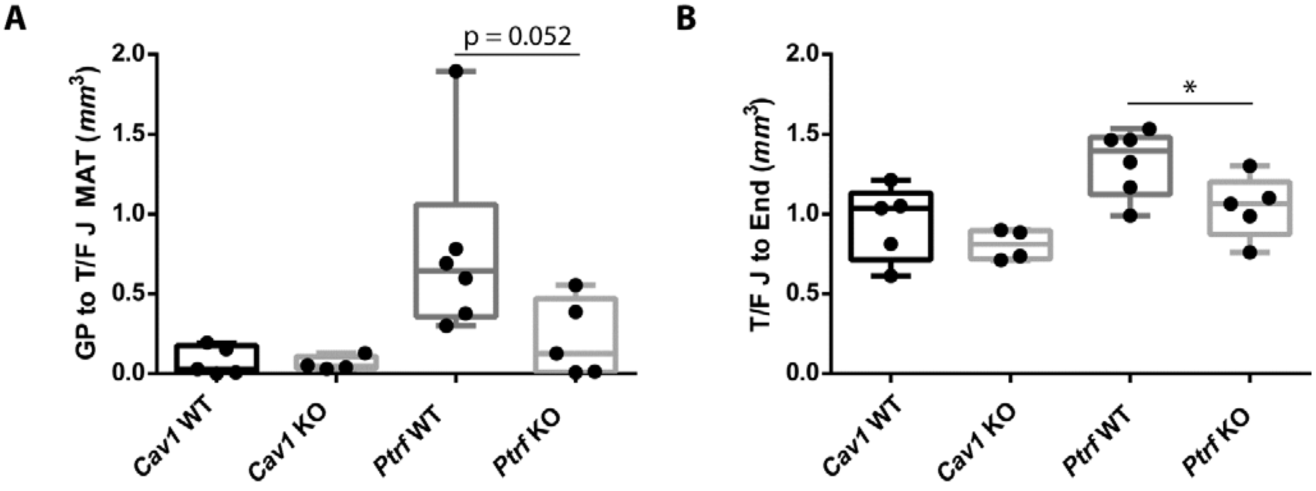

Supplementary Figure 4. Differential loss of MAT in female mice with knock-out of *Cav1* or *Ptrf*. (A,B) Region-specific quantification of tibial MAT volume by µCT (biological replicate N = 4-6 as indicated in the graph). Box plot center line represents median, box extends from the 25th to 75th percentile, whiskers indicate range. *(A) Non-parametric Mann-Whitney test, (B) two-tailed *t*-Test, $p<0.05$.

SUPPLEMENTARY FIGURE 5

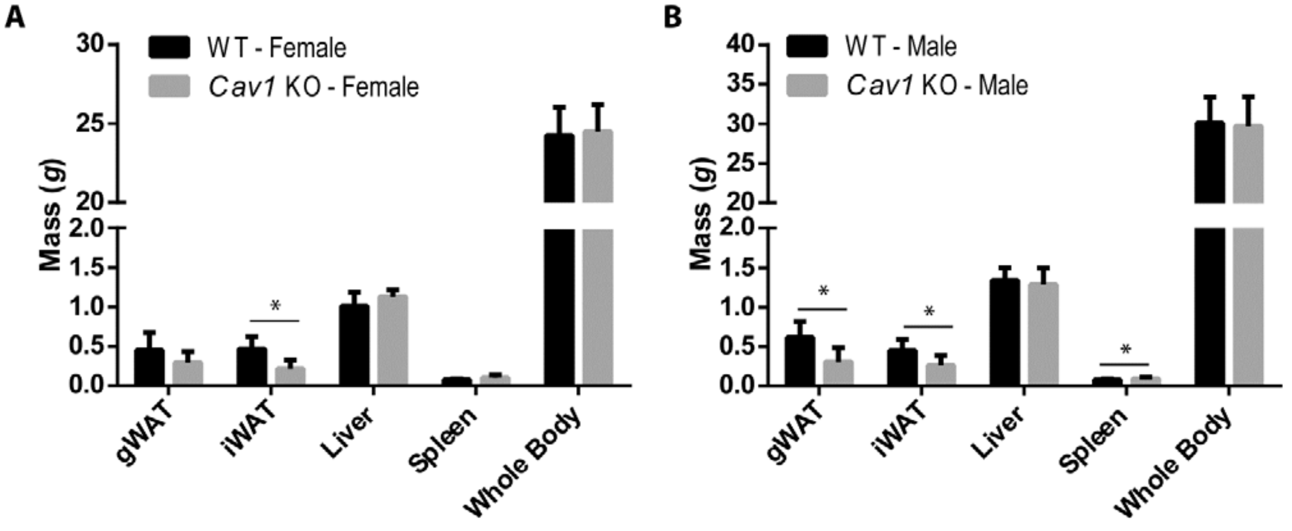

C

Proximal Tibial Metaphysis	Female WT	Female *Cav1* KO	Female P < 0.05	Male WT	Male *Cav1* KO	Male P < 0.05
Total Volume (*mm*^{3})	1.09 +/- 0.41	0.95 +/- 0.43		1.43 +/- 0.25	1.36 +/- 0.12	
Bone Volume Fraction (*%*)	37.8 +/- 8.3	44.9 +/- 8.7		41.9 +/- 5.2	42.8 +/- 7.7	
Trabecular Number (*mm*$^{-1}$)	5.61 +/- 0.68	5.93 +/- 0.58		6.25 +/- 0.55	6.74 +/- 0.66	
Trabecular Thickness (*mm*)	0.078 +/- 0.009	0.085 +/- 0.009		0.077 +/- 0.005	0.074 +/- 0.007	
Trabecular Spacing (*mm*$^{-1}$)	0.17 +/- 0.03	0.15 +/- 0.03		0.14 +/- 0.02	0.13 +/- 0.02	
Bone Mineral Content (*mgHA*)	332 +/- 76	400 +/- 71		356 +/- 38	354 +/- 65	
Marrow Adipose Tissue (*mm*^{3})	0.050 +/- 0.059	0.036 +/- 0.025		0.039 +/- 0.063	0.029 +/- 0.053	

D

	Tibia Mid-Cortical Morphology	Female WT	Female *Cav1* KO	Female P < 0.05	Male WT	Male *Cav1* KO	Male P < 0.05
3D	Total Volume (*mm*^{3})	0.35 +/- 0.03	0.37 +/- 0.05		0.41 +/- 0.05	0.44 +/- 0.06	
	Relative Cortical Volume (*%*)	63.1 +/- 3.9	65.4 +/- 1.6		66.0 +/- 3.7	63.5 +/- 3.6	
	Bone Mineral Content (*mgHA*)	825 +/- 54	854 +/- 21		848 +/- 46	812 +/- 56	
2D	Total Area (*mm*^{2})	1.02 +/- 0.08	1.08 +/- 0.13		1.18 +/- 0.14	1.24 +/- 0.19	
	Cortical Bone Area (*mm*^{2})	0.64 +/- 0.06	0.70 +/- 0.09		0.78 +/- 0.11	0.79 +/- 0.10	
	Cortical Thickness (*mm*)	0.22 +/- 0.02	0.24 +/- 0.02		0.25 +/- 0.03	0.24 +/- 0.02	

Supplementary Figure 5. Body mass, tissue mass, and bone parameters of wild-type and *Cav1* knock-out mice. Male and female mice at 16 weeks of age. **(A,B)** Body and tissue mass (biological replicate N = 8 (Female); 9 (Male KO); 14 (Male WT)). Gonadal white adipose tissue (gWAT), inguinal WAT (iWAT). **(C)** Quantification of trabecular parameters and MAT volume in the proximal tibial metaphysis (biological replicate N = 8 (Female); 13 (Male WT); 9 (Male KO)). **(D)** Quantification of cortical parameters at the mid-diaphysis (biological replicate N = 8 (Female); 13 (Male WT); 9 (Male KO)). Presented as mean ± standard deviation. *Two-tailed t-Test, p<0.05.

SUPPLEMENTARY FIGURE 6

A

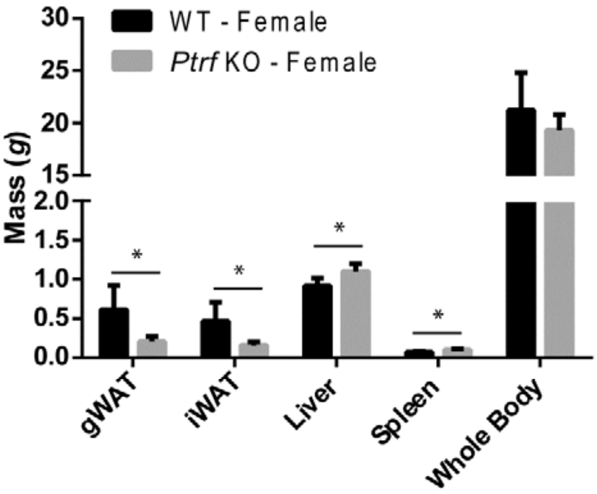

B

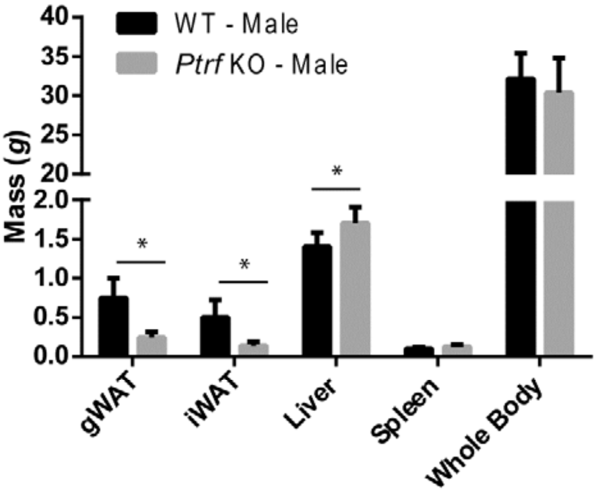

C

		Female			Male		
	Tibia Mid-Cortical Bone	WT	*Ptrf* KO	P < 0.05	WT	*Ptrf* KO	P < 0.05
3D	Total Volume (*mm^3*)	0.43 +/- 0.03	0.43 +/- 0.01		0.54 +/- 0.02	0.57 +/- 0.05	
	Bone Volume Fraction (*%*)	53.2 +/- 3.1	55.0 +/- 2.3		54.1 +/- 1.0	55.6 +/- 1.1	
	Bone Mineral Content (*mgHA*)	664 +/- 54	694 +/- 30		662 +/- 16	694 +/- 22	*
2D	Total Area (*mm^2*)	1.22 +/- 0.08	1.25 +/- 0.02		1.51 +/- 0.09	1.54 +/- 0.16	
	Bone Area (*mm^2*)	0.65 +/- 0.04	0.69 +/- 0.03		0.84 +/- 0.06	0.87 +/- 0.09	
	Cortical Thickness (*mm*)	0.19 +/- 0.01	0.20 +/- 0.01		0.21 +/- 0.02	0.22 +/- 0.01	

Supplementary Figure 6. Body mass, tissue mass, and cortical parameters of wild type and *Ptrf* knock-out mice. (A) Tissue and body mass from female mice at 12-15 weeks of age (biological replicate N = 5). **(B)** Tissue and body mass from male mice at 12-16 weeks of age (biological replicate N = 7 (WT); 8 (KO)). **(C)** Quantification of cortical parameters at the mid-tibial diaphysis in male and female mice at 16-17 weeks of age (biological replicate N = 5 (Female KO); 6 (Female WT); 7 (Male KO); 9 (Male WT)). Gonadal white adipose tissue (gWAT), inguinal WAT (iWAT). Presented as mean ± standard deviation. *Two-tailed t-Test, $p<0.05$.

SUPPLEMENTARY TABLE 1

Transcript	NCBI RefSEQ	Primers	
Scd1	NM_139192	Forward	CTCAGCGCTGGGAAAGTG
		Reverse	GAACTGGAGATCTCTTGGAGCA
Scd2	NM_031841.1	Forward	GCAGATGTTCGCCCTGAAATTA
		Reverse	CAAATATGCAAAGAGGCAGGTGTAG
Fads1	NM_053445.2	Forward	CACTACGCTGGTCAGGATGC
		Reverse	AGTGAGCGCCTTATTCTTGGT
Fads2	NM_031344.2	Forward	GACATTTCCAGCACCATGCG
		Reverse	TTCTTGCCATACTCGAGGGG
Fads3	NM_173137.1	Forward	CCCTGGTCAAGGCATTCTGT
		Reverse	AGACTTCTTCAGGGACCCGAT
Gpam	NM_017274.1	Forward	CTGTCTGGGGATAGCTTTGCT
		Reverse	ACAAAACATGAGCTGTATCCTTGA
Tbp	NM_001004198.1	Forward	AGTGCCAAGTGTGAGCCTCT
		Reverse	ACAGTGATGTGGGGACAAAACG
Srebf1	NM_001276707.1	Forward	CCATGGACGAGCTACCCTTC
		Reverse	AGCATGTCTTCGATGTCGGT
Pparg	NM_013124.3	Forward	AACTCTGGGAGATCCTCCTGT
		Reverse	ATGGTAATTTCTTGTGAAGTGCTCA
*Cebp*a	NM_001287577.1	Forward	TGCGCAAGAGCCGAGATAAA
		Reverse	GCGGTCATTGTCACTGGTCA
*Cebp*b	NM_024125.5	Forward	TTCCTTTCCGACCTCTTCGC
		Reverse	CACGTAACCGTAGTCGGACG

Supplementary Table 1. Primer Sequences. Rat primer sequences for qPCR.
